# Diversified Polyketides With Anti-inflammatory Activities From Mangrove Endophytic Fungus *Daldinia eschscholtzii* KBJYZ-1

**DOI:** 10.3389/fmicb.2022.900227

**Published:** 2022-05-10

**Authors:** Guisheng Wang, Zhenhua Yin, Senye Wang, Yilin Yuan, Yan Chen, Wenyi Kang

**Affiliations:** ^1^National R&D Center for Edible Fungus Processing Technology, Henan University, Kaifeng, China; ^2^Joint International Research Laboratory of Food and Medicine Resource Function, Kaifeng, China; ^3^Kaifeng Key Laboratory of Functional Components in Health Food, Kaifeng, China

**Keywords:** mangrove endophytic fungus, *Daldinia eschscholtzii*, anti-inflammatory activity, NF-κB, MAPK

## Abstract

In total, five new polyketide derivatives: eschscholin B (**2**), dalditone A and B (**3** and **4**), (1*R*, 4*R*)-5-methoxy-1,2,3,4-tetrahydronaphthalene-1,4-dio (**5**), and daldilene A (**6**), together with 10 known as analogs (**1**, **7**–**15**) were isolated from the mangrove endophytic fungus *Daldinia eschscholtzii* KBJYZ-1. Their structures and absolute configurations were established by extensive analysis of NMR and HRESIMS spectra data combined with ECD calculations and the reported literature. Compounds **2** and **6** showed significant cell-based anti-inflammatory activities with IC_50_ values of 19.3 and 12.9 μM, respectively. In addition, western blot results suggested that compound **2** effectively inhibits the expression of iNOS and COX-2 in LPS-induced RAW264.7 cells. Further molecular biology work revealed the potential mechanism of **2** exerts anti-inflammatory function by inactivating the MAPK and NF–κB signaling pathways.

## Introduction

Mangrove endophytic fungi have proven to be a promising source of novel chemical backbones and bioactive metabolites owing to extreme environments (tidal flooding, high salinity, anaerobic soil, and high temperature) of mangroves (Chen S. et al., 2022; Chen Y. et al., 2022). *Daldinia eschscholtzii* is an endophytic fungus isolated commonly from mangrove plants (Yang et al., 2017). The diverse bioactivity metabolites, including tetralones (Liao et al., 2019a), lactones (Kongyen et al., 2015), naphthoquinones (Wutthiwong et al., 2021), chromones (Barnes et al., 2016), and polyphenols (Zhang et al., 2016), have attracted much attention. For instance, naphthoquinones 5-hydroxy-2-methoxy-6,7-dimethyl-1,4-naphthoquinone form *D. eschscholtzii* HJ004 showed antibacterial activity (Liao et al., 2019b), and chromones 5-hydroxy-8-methoxy-2-methyl-4H-chromen-4-one from *D. eschscholtzii* GsE13 showed phytotoxicity (Flores-Reséndiz et al., 2021).

**Figure 1:**
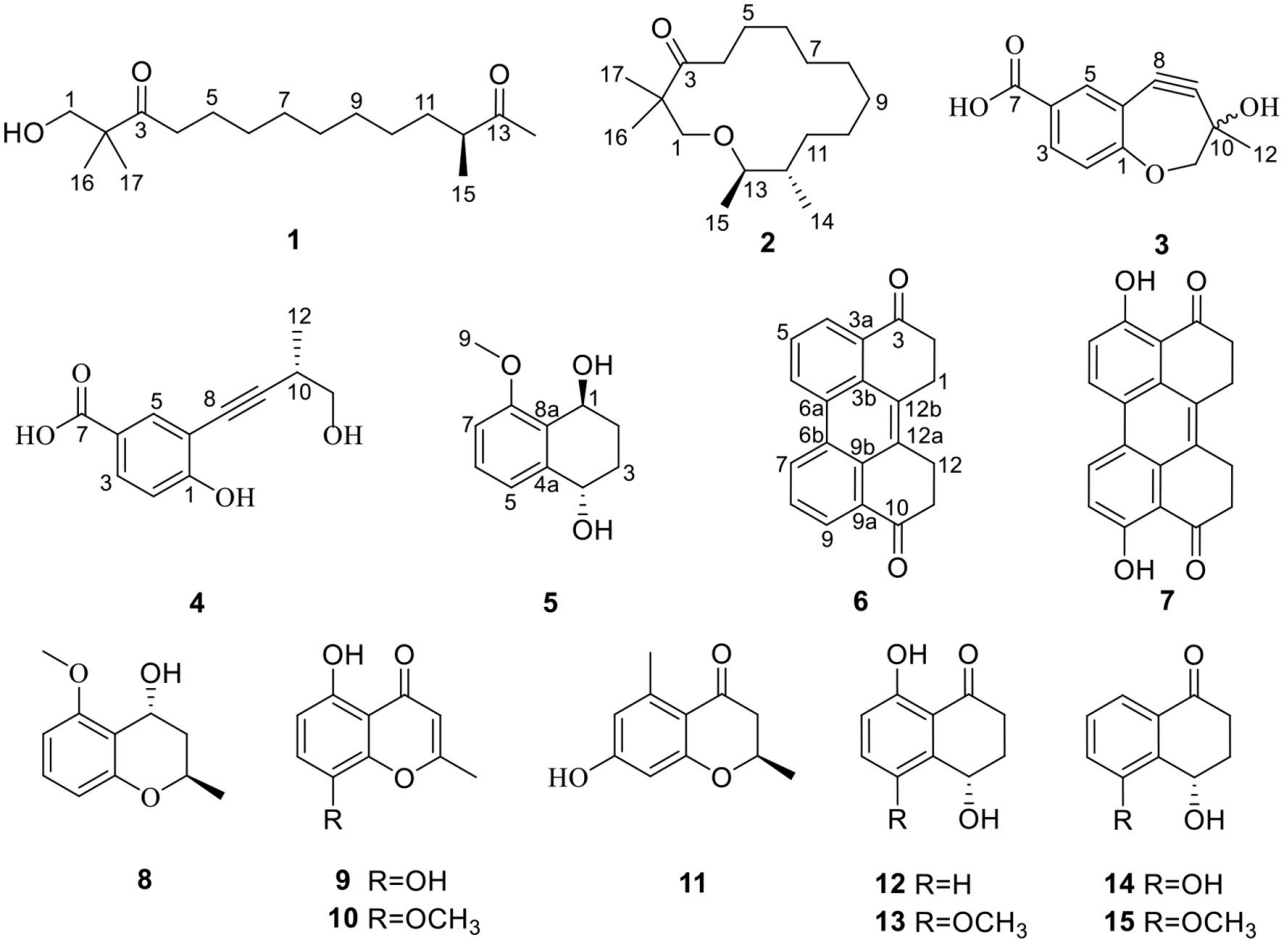
The structures of **1-15**.

It is well known that excessive inflammation could lead to tissue damage, loss of function, and many more related diseases, such as arthritis, systemic lupus erythematosus, ulcerative colitis, and cancer (Zhang Y. et al., 2021). The most often used therapeutic medicines, such as non-steroidal anti-inflammatory drugs (NSAIDs), have been shown to significantly reduce prostaglandin production by reducing the activity of cyclooxygenase (COX) enzymes (Bindu et al., 2020). Whereas, various side effects might be caused by NSAIDs, including gastrointestinal mucosal injury, liver, and kidney toxicity (Wang et al., 2020). As a result, the discovery of new anti-inflammatory medications has become an unavoidable trend. The metabolites from mangrove endophytic fungus were the key sources of the anti-inflammatory lead compounds due to their novel structure, low toxicity, and significant inhibitory effect (Chen et al., 2021). As part of our continuing investigation into searching for novel anti-inflammatory natural compounds derived from mangrove endophytic fungi, a fungus *D. eschscholtzii* KBJYZ-1, which was isolated from *Pluchea indica* Less., aroused our interest because the ethyl acetate extract of the fungal culture displayed excellent anti-inflammatory activity. As a result, five new compounds (**2**–**6**) and ten known compounds (**1**, **7**–**15**) were isolated (Figure 1). The anti-inflammatory activity of all isolated compounds was evaluated by the lipopolysaccharides (LPSs) induced NO production in RAW264.7 macrophages. Moreover, the potential anti-inflammatory mechanism of **2** has been investigated.

## Materials and Methods

### General Experimental Procedures

Specific optical rotation was measured on a PerkinElmer 341 instrument at 25°C. Cary 5000 spectrophotometer was used to record UV spectra in MeOH. ECD data were obtained by Model 420SF CD spectrometer (Aviv Biomedical Inc). In KBr discs, IR spectra were obtained using Fourier infrared IS50 spectrometer. All NMR experiments were performed at room temperature on a Bruker AVANCE 500 spectrometer using the signals of residual solvent protons (CDCl_3_: δ_H_ 7.26; CD_3_OD: δ_H_ 3.31) and carbons (CDCl_3_: δ_C_ 77.1; CD_3_OD: δ_C_ 49.2). HRESIMS spectra were tested by Waters TQ-XS mass spectrometer. Column chromatography (CC) was conducted by silica gel (200–300 mesh, Yantai Huiyou Silica gel company) and Sephadex LH-20 (CHCl_2_/MeOH, *v/v* 1:1) (Pharmacia Sweden). On silica gel plates, thin layer chromatography (TLC) was conducted (GF 254 Silica gel Thin Layer Plate Yantai Huiyou Silica company). The Typical Culture Preservation Committee Cell Bank, China provided RAW264.7 cells; the fetal bovine serum (FBS) was obtained by Gibco; ProCell provided Dulbecco's modified Eagle's medium (DMEM); Sigma supplied LPS and _L_-NMMA; Shanghai Beyotime Biotechnology supplied the NO kit. Thermo Fisher Scientific (Shanghai, China) provided the primers for iNOS. Cell Signaling (Beverly, MA, USA) supplied all the antibodies.

### Fungal Material

The strain KBJYZ-1 was isolated from the root of *Pluchea indica* Less., which was collected in July 2020 from Zhanjiang Mangrove National Nature Reserve in Guangdong Province, China. Fungal identification was carried out using molecular biological methods to identify fungal species by DNA amplification and ITS sequences (Chen et al., 2018). BLAST analysis showed that this sequence had the highest homology with 100% to the sequence of *Daldinia eschscholtzii* (compared with MW081312.1). Sequence data of the strain is deposited at GenBank with accession no. OM267787. The fungus was preserved at Henan University, China.

### Fermentation Extraction and Isolation

The fungus was cultured on solid rice medium (100 numbers of 1,000 ml Erlenmeyer flasks, each containing 120 g rice and 75 ml of 0.3% seawater) at room temperature for one month under static condition. After fermentation, the mediums were extracted with MeOH three times. The organic phase was concentrated under reduced pressure to yield a total residue of 78.6 g. Moreover, a silica gel column was used for chromatography (CC) was used with petroleum ether/ethyl acetate gradient elution from 10:0 to 2:8, to obtain initial ten fractions (Fr.1–Fr.10) were obtained. Fr.2 (200.6 mg) was separated by Sephadex LH-20 to obtain **8** (3.2 mg). The subfraction Fr.2.1 (60.3 mg) was further subject to silica gel CC (CH_2_Cl_2_:PE *v*/*v*, 2:1) to obtain **1** (2.6 mg). The subfraction Fr.2.1.4 and Fr.2.3 were pooled and purified by Sephadex LH-20 CC (CH_2_Cl_2_/MeOH *v*/*v*, 1:1) to get **5** (2.1 mg) and **6** (3.3 mg), respectively. In total, **7** (4.1 mg) and **9** (2.3 mg) were obtained from subfraction Fr.2.4 (40.9 mg) which was purified by silica gel CC (CH_2_Cl_2_). Fraction Fr.3 (100.3 mg) was separated by Sephadex LH-20 CC (CH_2_Cl_2_/MeOH *v*/*v*, 1:1) to get two subfraction Fr.3.1-3.2. Subfraction Fr.3.2 (80.3 mg) was further purified to obtain six subfractions (3.2.1–3.2.6). **2** (2.2 mg) was obtained by purification Fr.3.2.6 (6.5 mg) using sephadex LH-20 CC (CH_2_Cl_2_/MeOH *v*/*v*, 1:1). Fr.4 (450.0 mg) was purified and fractionated into four subfractions (4.1–4.4) by Sephadex LH-20 CC (CH_2_Cl_2_/MeOH *v*/*v*, 1:1). Fr.4.1 (125.3 mg) was again fractionated using silica gel CC (CH_2_Cl_2_/MeOH *v*/*v*, 125:1~80:1), and subfractions Fr.4.1.2 (15.3 mg) and Fr.4.1.4(10.3 mg) were purified by Sephadex LH-20 CC (CH_2_Cl_2_/MeOH *v*/*v*, 1:1) to obtain **13** (1.8 mg) and **15** (1.3 mg), respectively. Fr.5 (200.3 mg) was purified by Sephadex LH-20 CC (CH_2_Cl_2_/MeOH *v*/*v*, 1: 1) to yield **10** (5 mg) and **12** (4.3 mg), and other subfractions (5.1–5.8). Fr.5.3 (20.4 mg) was separated into four subfractions (Fr.5.3.1–Fr.5.3.4) using silica gel CC (CH_2_Cl_2_/MeOH *v*/*v*, 100:1, 95:1, 90:1, 80:1), and subfractions Fr.5.3.2 furnished **11**(3.5 mg). Subfractions Fr.5.3.4 purified by Sephadex LH-20 furnished **14**. Fr.6 (585.0 mg) was purified by Sephadex LH-20 furnished to give five fractions (6.1–6.5). Fraction of Fr.6.4 (221.0 mg) purified by silica gel CC resulted four fractions (6.4.1–6.4.4), purification of subfraction Fr.6.4.2 (15.3 mg) and Fr.6.4.3 (10.9 mg) by Sephadex LH-20 furnished **3** (4.2 mg) and **4** (3.5 mg), respectively.

Eschscholin B (**2**): yellow oil; [α] = −15.1 (*c* 0.26, MeOH); UV (MeOH) λ_max_ (log ε): 210 (1.68) nm; IR (KBr) ν_max_: 2,935, 2,856, 2,355, 1,702, 1,464, 1,378, 1,284, 1,053 cm^−1^; ^1^H and ^13^C NMR (CDCl_3_) data (Table 1); HRESIMS *m/z* 269.2470 [M + H]^+^ (calcd for C_17_H_33_O, 269.2468).

**Table 1:** ^1^H and ^13^C NMR data of **2** in CDCl_3_.

**No**.	**δ_C_**	**δ_H_ [mult, *J* (Hz)]**	**No**.	**δ_C_**	**δ_H_ [mult, *J* (Hz)]**
1	69.3, CH_2_	3.43, s	10	27.2, CH_2_	1.15, overlap
2	49.0, C		11	29.4, CH_2_	1.15, overlap
3	216.9, C		12	39.6, CH	1.29, m
4	37.3, CH_2_	2.38, t (7.3)	13	71.1, CH	3.55, td (6.3, 10.7)
5	23.5, CH_2_	1.43, dd (7.0, 14.0)	14	14.2, CH_3_	0.75, d (6.8)
6	29.8, CH_2_	1.30, m	15	20.0, CH_3_	1.0, d (6.3)
7	29.4, CH_2_	1.15, overlap	16	21.5, CH_3_	1.02, s
8	29.1, CH_2_	1.15, overlap	17	21.5, CH_3_	1.02, s
9	32.4, CH_2_	1.15, overlap			

Dalditone A (**3**): yellow solid; [α] = + 0.01 (*c* 0.12, MeOH); UV (MeOH) λ_max_ (log ε): 266 (1.77), 205 (1.89) nm; IR (KBr) ν_max_: 3,381, 2,928, 2,965, 2,130, 1,760, 1,650, 1,463, 1,064 cm^−1^; ^1^H NMR (MeOH-*d*_4_) data (Table 1); ^13^C NMR (MeOH-*d*_4_) data (Table 2); HRESIMS *m/z* 243.0627 [M + Na]^+^ (calcd for C_12_H_10_O_4_Na, 243.0620).

**Table 2:** ^1^H and ^13^C NMR data of **3** and **4** in MeOH-*d*_4_.

**No**.	**3**		**4**	
	**δ_C_, type**	**δ_H_, mult (*J* in Hz)**	**δ_C_, type**	**δ_H_, mult (*J* in Hz)**
1	163.2, C		161.7, C	
2	116.3, CH	6.88, d (8.6)	114.7, CH	6.85, d (8.6)
3	132.7, CH	7.83, dd (2.1, 8.6)	130.7, CH	7.79, dd (2.2, 8.6)
4	123.7, C		123.4, C	
5	136.3, CH	7.98, d (2.1)	134.9, CH	7.92, d (2.2)
6	111.3, C		110.8, C	
7	169.6, C		168.1, C	
8	80.0, C		76.3, C	
9	97.4, C		96.3, C	
10	69.8, C	3.56, dd (6.5, 10.5)	29.8, CH	2.85, dd (6.7, 13.5)
11a	71.1, CH_2_	3.61, s	65.8, CH_2_	3.65, dd (6.5, 10.5)
11b				3.56, dd (6.5, 10.5)
12	26.2, CH_3_	1.53, s	16.2, CH_3_	1.27, d (1.9)

Dalditone B (**4**): yellow solid; [α] = + 10.5 (*c* 0.33, MeOH); UV (MeOH) λ_max_ (log ε): 260 (1.23), 224 (1.84), 201 (1.71) nm; IR (KBr) ν_max_: 3,288, 2,928, 2,867, 2,200, 1,671, 1,556, 1,460, 1,299, 1,113, 1,039 cm^−1^; ^1^H and ^13^C NMR (MeOH-*d*_4_) data (Table 2); HRESIMS *m/z* 219.0649 [M – H]^−^ (calcd for C_12_H_11_O_4_, 219.0643).

(1*R*, 4*R*)-5-methoxy-1,2,3,4-tetrahydronaphthalene-1,4-dio (**5**): colorless solid; [α] = +23.2 (*c* 0.60, MeOH); UV (MeOH) λ_max_ (log ε): 254 (1.80), 210 (1.44) nm; IR (KBr) ν_max_: 3,389, 3,004, 2,945, 1,728, 1,580, 1,463, 1,269, 1,018, 993, 754 cm^−1^; ^1^H and ^13^C NMR (CDCl_3_) data (Table 3); HRESIMS *m/z* 194.0498 [M – H]^−^ (calcd for C_11_H_13_O_3_, 194.0490).

**Table 3:** ^1^H and ^13^C NMR data of **5** and **6** in CDCl_3_.

**No**.	**5**		**No**.	**6**	
	**δ_C_**	**δ_H_ [mult, *J* (Hz)]**		**δ_C_**	**δ_H_ [mult, *J* (Hz)]**
1	63.2, CH	5.06, t (4.7)	1, 12	25.4, CH_2_	3.54, t (4.7)
2a	25.7, CH_2_	1.88, m	2, 11	37.7, CH_2_	3.05, m
2b		2.27, m	3, 10	198.3, C	
3a	27.7, CH_2_	1.80, m	4, 9	126.4, CH	8.95, d (8.3)
3b		2.19, m	5, 8	126.5, CH	7.79, t (7.6)
4	67.7, CH	4.79, m	6, 7	128.7, CH	8.38, d (7.4)
4a	139.9, C		3a, 9a	129.2, C	
5	120.9, CH	7.07, d (7.8)	6a, 6b	128.7, C	
6	129.0, CH	7.29, t (8.0)	12a, 12b	131.2, C	
7	109.7, CH	6.85, d (8.2)	3b, 9b	129.0, C	
8	159.5, C				
8a	126.7, C				
9	55.5, CH_3_	3.89, s			

Daldilene A (**6**): yellow solid; UV (MeOH) λ_max_ (log ε): 258 (1.5), 206 (2.1) nm; IR (KBr) ν_max_: 2,917, 2,949, 1,765, 1,650, 1,193, 1,068 cm^−1^; ^1^H and ^13^C NMR (CDCl_3_) data (Table 3); HRESIMS *m/z* 287.1054 [M + H]^+^ (calcd for C_20_H_15_O_2_, 287.1051).

### ECD Calculations

The ECD calculations were performed according to the method described previously (Chen et al., 2019). The conformers of compounds **1**, **2**, **4**, and **5** were optimized using DFT calculations at B3LYP/6-31g (d) level in MeOH. Then, ECD calculations were conducted using time-dependent density functional theory (TD-DFT) at B3LYP/DGDZVP, PBEPBE/6-311+G, B3LYP/6-31G, and B3LYP/6-311G levels, respectively.

### Anti-inflammatory Assay

#### Cell Culture

RAW264.7 cells were cultured in Dulbecco's modified Eagle's medium (DMEM) containing 10% fetal bovine serum, 100 U/ml penicillin, and 100 μg/ml streptomycin at 37°C with 5% CO_2_.

#### Cell Viability Assay

The cell viability was evaluated using the MTT assay as described previously (Niu et al., 2021). Briefly, RAW264.7 cells (5 × 10^4^ cells/well) with logarithmic growth were inoculated in 96-well plates for 12 h at 37°C with 5% CO_2_. Cells were treated with different concentrations of _L_-NMMA or the test compounds (10, 20, 30, 40, and 50 μM) and LPS (1 μg/ml) for 24 h. Then, approximately 10 μl of MTT (0.5 mg/ml) was added to each well and incubated for 4 h at 37°C. After completion of the post-incubation, the absorbance was measured at 490 nm.

#### Measurement of NO Production

RAW264.7 cells were inoculated in 96-well plates and incubated for 14 h at 37°C. Period, different concentrations of L-NMMA or the test compound were added, and stimulated with LPS (1 μg/ml) for 24 h. The levels of NO were measured according to the instructions of the manufacturer. The absorbance was measured at 540 nm.

#### Western Blot

Briefly, RAW264.7 cells (1 × 10^6^ cells/well) were inoculated into the 6-well plates and incubated with 2 ml DMEM at 37°C. The spent cell culture medium was discarded when the cell fusion reached about 70–80%. Then, cells were stimulated with compounds (25, 12.5, and 6.25 μM), and incubated for 24 h. Western blot was carried out and the assay was done as described previously (Niu et al., 2021). Blots were visualized using enhanced chemiluminescence (ECL) detection kits and analyzed using the Image J software.

### Statistical Analysis

All the experiments were repeated at least three times and statistical analyses were evaluated using the GraphPad Prism 7 program. The data were expressed as a means ± SD. *p* < 0.05 indicates statistical significance. A one-way ANOVA analysis was used to determine statistical significance.

## Results and Discussion

### Structure Elucidation

**Figure 2:**
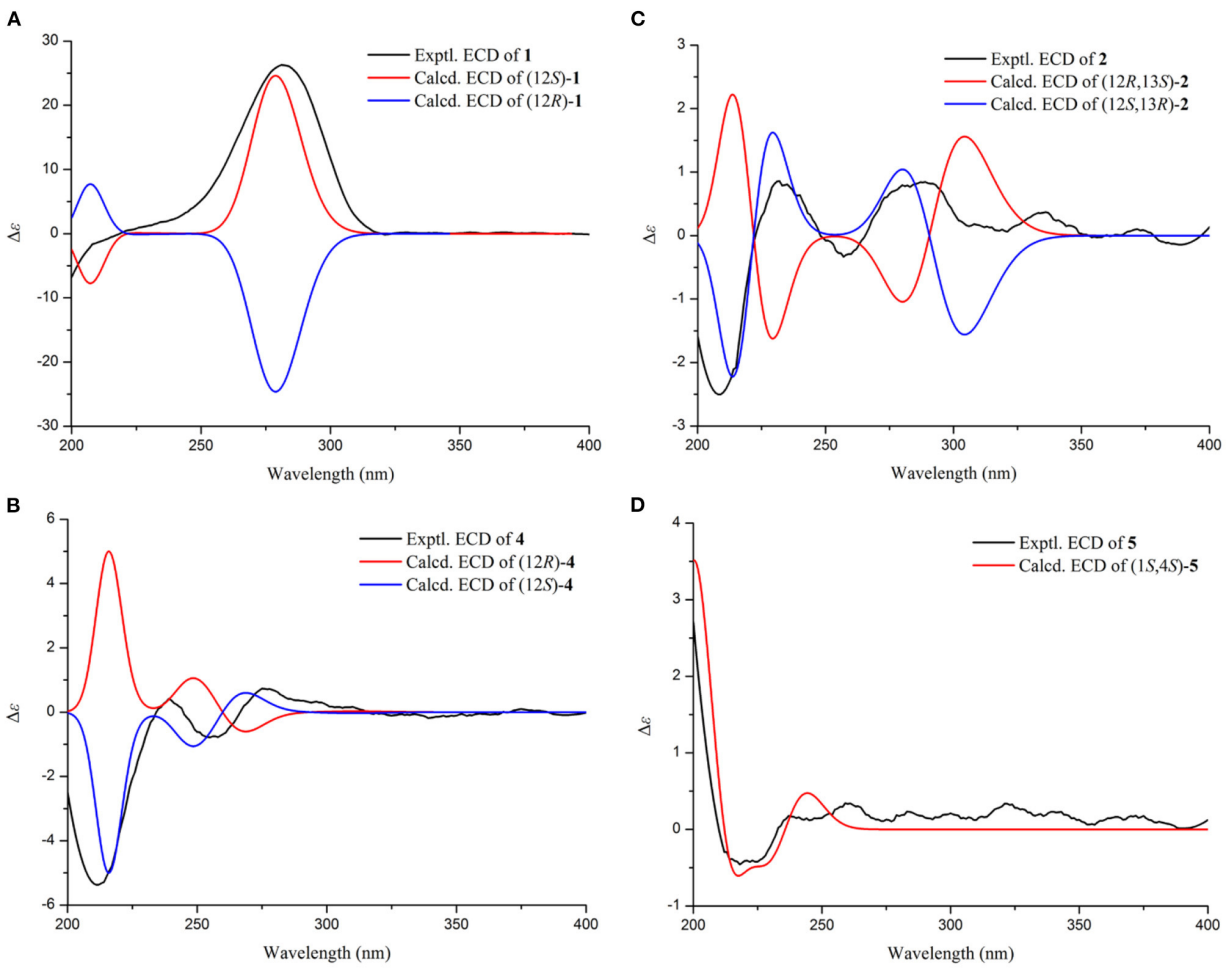
Experimental and calculated ECD curves for **1 (A)**; **2 (B)**; **4 (C)**; **5 (D)**.

Compound **1** was identified as eschscholin A (Liu et al., 2017) by comparing the ^1^H and ^13^C NMR data (Supplementary Table S1). Here, the absolute configuration of 12*S* was first determined by ECD calculation (Figure 2).

**Figure 3:**
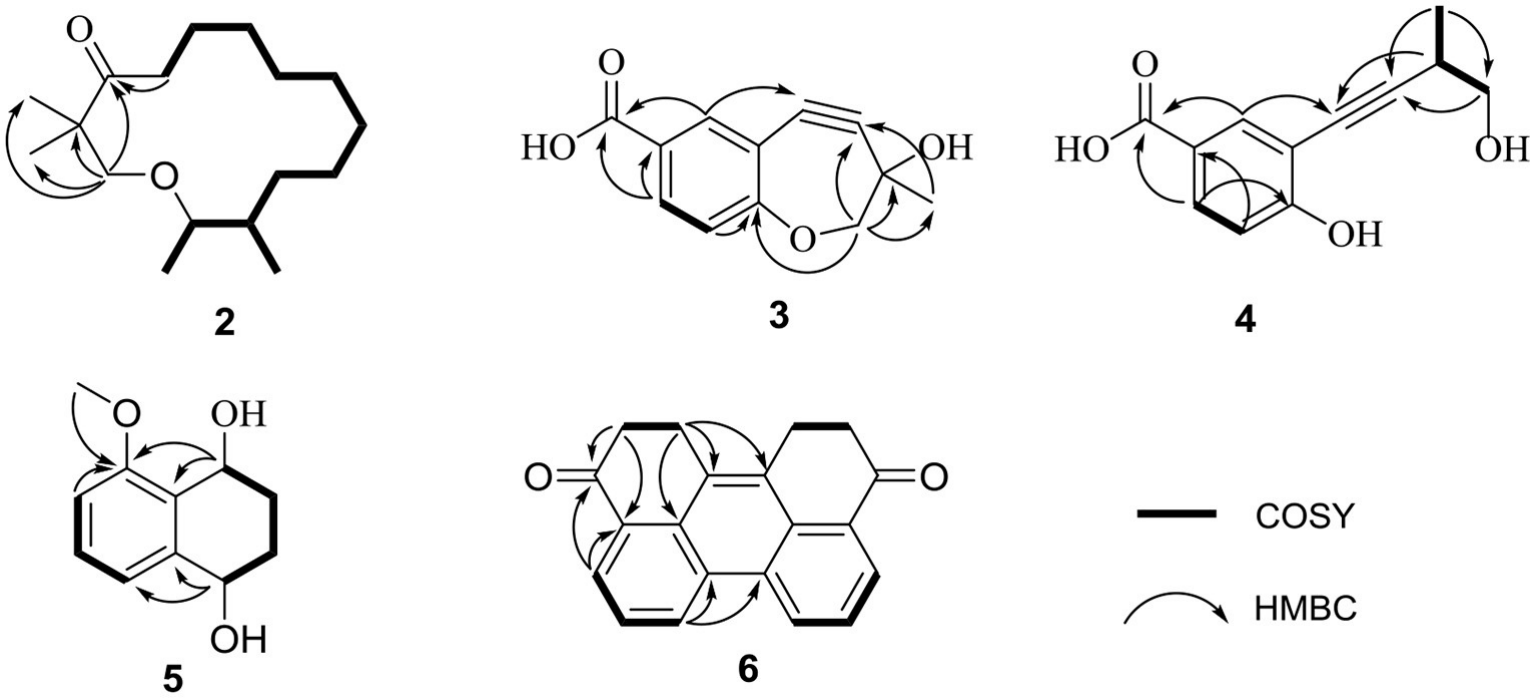
Key HMBC and COSY correlations of **2-6**.

**Figure 4:**
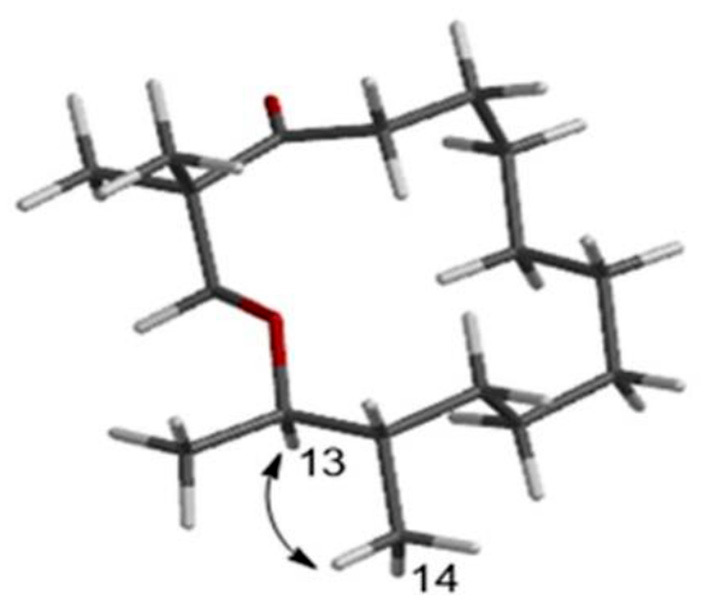
NOESY correlation of **2**.

Compound **2**, a yellow oil, had a molecular formula of C_17_H_32_O_2_. As established by high-resolution electrospray ionization mass spectrometry (HRESIMS), it showed two degrees of unsaturation. The ^1^H NMR spectrum (Table 1), provided signals for four methyls at δ_H_ 0.75 (d, *J* = 6.8 Hz, H_3_-14), 0.7 (d, *J* = 6.8 Hz, H_3_-15), 1.02 (s, H_3_-16), and 1.02 (s, H_3_-17); one oxygenated methylene at δ_H_ 3.43 (s, H_2_-1), 2.38 (t, *J* = 7.3 Hz, H_2_-15); an oxygenated methine group at δ_H_ 3.55 (td, *J* = 6.3 Hz, 10.3 Hz, H-13). ^13^C NMR (Table 1) and HSQC spectra data of **2** exhibited 17 carbon signals, including four methyls, ten methylenes, two methines, and one carbonyl carbon. Moreover, the spin system of H_2_-4/H_2_-5/H_2_-6/H_2_-7/H_2_-8/H_2_-9/H_2_-10/H_2_-11/H-12(/H_3_-14)/H-13/H_3_-15 from COSY data (Figure 3), together with the HMBC correlations (Figure 3) from H_3_-16 to C-2 and C-1, from H_3_-17 to C-2 and C-3, from H_2_-4 to C-3, established the preliminary structure. Finally, except for a carbonyl group, the remaining indices of hydrogen deficiency were determined as 14-membered macrocycle. Comparing the NMR data indicated the structure of **2** was a resemblance to eschscholin A (Liu et al., 2017). Thus, the structure of **2** was established as exhibited in Figure 1. The relative configuration of **2** was confirmed by the NOESY correlation of H-13/H_3_-14, together with the large coupling constant *J*_H−12, H−13_ = 10.7 Hz (Figure 4). Furthermore, the absolute configuration was confirmed by the ECD calculation. The identical experimental and calculated ECD curves (Figure 2) assigned the 12*S*, 13*R* configuration of **2**.

Compound **3** was obtained as a yellow solid. The molecular formula was determined as C_12_H_10_O_4_, based on the HRESIMS data. The ^1^H NMR spectrum (Table 2) provided signals for one methyl δ_H_ 1.53 (s, H_3_-12), one oxygenated methylene δ_H_ 3.61 (s, H_2_-11), three methines δ_H_ 6.88 (d, *J* = 8.6 Hz, H-2), 7.83 (dd, *J* = 2.1 Hz, 8.6 Hz, H-3), 7.98 (d, *J* = 2.1 Hz, H-5). The ^13^C NMR (Table 2) and HSQC spectra displayed 12 carbons, including one methyl, one methylene, six sp^2^ carbons, two sp carbons, and one carboxyl carbon. The HMBC correlations (Figure 3) from H-5 to C-8, from H_2_-11 to C-9, from H_3_-12 to C-9, C-10, and C-11, together with the chemical shift at C-8 (δ_C_ 80.0) and C-9 (δ_C_ 97.6), supported that the alkynyl group is located at C-6. Furthermore, the weak HMBC correlation from H_2_-11 to C-1 confirmed that C-11 and C-1 were connected by an oxygen atom. Compound **3** was determined to be the scalemic mixture as shown by the flat ECD spectra and tiny specific rotation value. The chiral-phase resolution under various circumstances was unsuccessful.

Compound **4**, a yellow solid, its molecular formula determined to be C_12_H_12_O_4_ by the HRESIMS, and indicated seven degrees of unsaturation. Comparing the NMR data (Table 2) disclosed a similar structure of **3** and **4**, except for the absence of the hydroxy at C-10 in **4**. The spin system of H_3_-12/H-10/H_2_-11 was observed from the COSY spectrum (Figure 3). The HMBC correlation (Figure 3) from H-10 to C-8 further confirmed the deduction. In addition, the HMBC correlation and HRESIMS supported that the ether bond between C-11 and C-1 was fractured. The 12*S* configuration was confirmed by the identical experimental and ECD calculation curves (Figure 2).

Compound **5**, a colorless solid, had a molecular formula of C_11_H_14_O_3_ by HRESIMS, and showed five degrees of unsaturation. ^1^H NMR (Table 3) showed three aromatic signal peaks at δ_H_ 7.07 (d, *J* = 7.8 Hz, H-5), 7.29 (d, *J* = 8.0 Hz, H-6), 6.85 (d, 8.2 Hz, H-7), two oxygenated methines signal peak δ_H_ 5.06 (t, *J* = 5.1 Hz, H-1), 4.79 (m, H-4). Comparing the NMR data (Table 3) revealed that **5** and **12** (Talapatra et al., 1988) had a similar structure. Except in **5**, where the carbonyl group at C-1 was converted to a hydroxy group. The above conclusion was verified by the H-1/H-2/H-3/H-4 correlation from the COSY spectrum (Figure 3), combine with the HMBC correlations (Figure 3) from H-1 to C-8 and C-8a. While, according to the HMBC correlation from H_3_-9 to C-8, decided that methoxy was located in C-8. The absence of correlation of H-1 and H-4 in the NOESY spectrum showed the 1S^*^, 4S^*^ configuration of **5**. Thereafter, the identical test and calculated ECD curves (Figure 2) determined the absolute configuration of compound **5** as 1*S*, 4*S*.

Compound **6**, a yellow solid, its molecular formula was identified as C_20_H_14_O_2_, according to the HRESIMS. The ^1^H-NMR (Table 3) showed three aromatic protons at δ_H_ 8.95 (d, *J* = 8.3 Hz), 7.79 (t, *J* = 7.6 Hz), 8.38 (d, *J* = 7.6 Hz), two methylene peaks δ_H_ 3.54 (t, *J* = 4.7 Hz) and 3.05 (m). While the ^13^C NMR (Table 3) and HSQC spectra exhibited 20 carbons, including four methyls, six sp carbons, and the rest of the carbons, were quaternary carbon (including two carbonyls). Comparison of the NMR data (Table 3) of **6** and **7**, showed a similar structure for **6** and **7**, except for the absence of the hydroxyl group at C-4 and C-9 in **6**. The deduction was supported by the H-4/H-5/H-6 correlation from the COSY spectrum and the HMBC correlations (Figure 3) from H-4 to C-3 and C-3a. Thus, the structure of **6** was established.

In total, ten other known compounds were characterized as 4, 9-dihydroxy-1, 2, 11, 12- tetrahydroper-ylene-3,10-quinone (**7**) (Li et al., 2006), (2*R*,4*R*)-3,4-dihydro-5-methoxy-2-methyl-2*H*-1-benzopyran-4-ol (**8**) (Zheng et al., 2016), 4*H*-1-benzopyran-4-ne,5,8-dihydroxy-2-methyl (**9**) (Rao and Venkateswarlu, 1956), 5-hydroxy-8-methoxy-2-methyl-4*H*-1benzopy ran-4-one (**10**) (Sun et al., 2012), (2*R*) 7-hydroxy-2,5-dimethylc-hromone (**11**) (Konigs et al., 2010), (-)-regiolone (**12**) (Talapatra et al., 1988), (4*S*)-4,8-dihydroxy-5-methoxy-*a*-tetralone (**13**) (Machida et al., 2005), (4*S*)-4,5-dihydroxy-α-tetralone (**14**) (Liu et al., 2004), (4*S*)-naphthalenone-3,4-dihydro-4-hydroxy-5-methosy (**15**) (Yamamoto et al., 2003) by comparison of the spectroscopic data with the previous literature.

**Figure 5:**
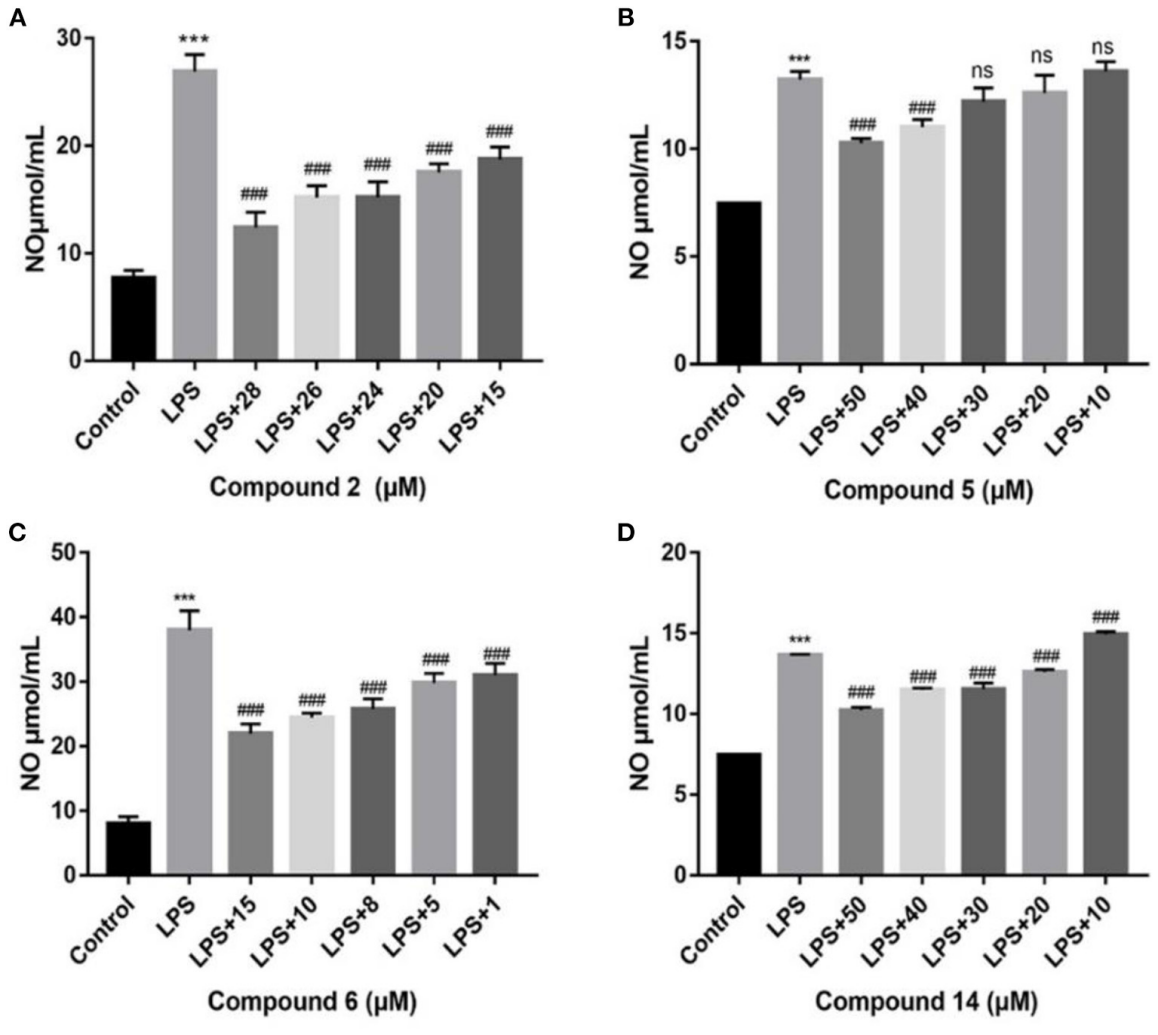
Influences of compounds on NO production for LPS-induced RAW264.7 cells. Compound **2 (A)**; compound **5 (B)**; compound **6 (C)**; compound **14 (D)**. Data rendered are the mean ± SD, *n* = 3. In comparison to the control, ***P < 0.001. In comparison to LPS, ^###^*P* < 0.001.

All compounds were assayed for the anti-inflammatory activities on mouse macrophage RAW264.7 cells. Compounds **2** and **6** showed a considerable inhibitory action, with IC_50_ values of 19.3 μM and 12.9 μM, respectively (positive control _L_-NMMA: 32.8 μM, Figure 5). Compounds **5** and **14**, showed weaker inhibitory activity compared with the positive control (Figure 5). Other compounds exhibited no inhibitory action (IC_50_ >50 μM). At the studied concentrations, none of the compounds were cytotoxic to RAW264.7 cells.

**Figure 6:**
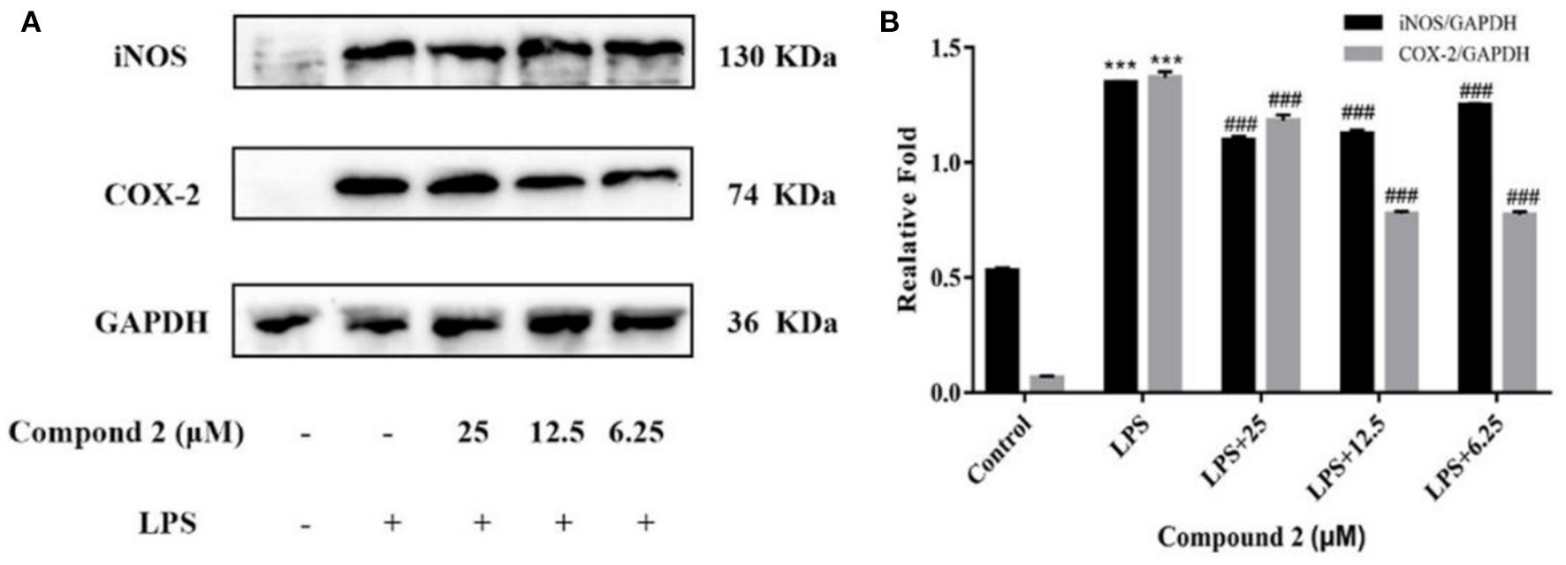
Influences of compound **2** on iNOS, COX-2, and GAPDH protein expression were detected by Western blotting **(A)**. The ratio of the content of iNOS/GAPDH and COX-2/GAPDH **(B)**. Data rendered are the mean ± SD, *n* = 3. In comparison to the control, ***P < 0.001. In comparison to the LPS group, ^###^*P* < 0.001.

Several inducible enzymes in macrophages were significantly up-regulated in the process of inducing inflammation. For example, rate-limiting enzymes responsible for NO production include iNOS. At the same time, inflammatory injury mainly stimulates monocytes, and macrophages induce COX-2 generation, which is a key link in triggering a subsequent inflammatory response. In conclusion, iNOS and COX-2 were considered valuable targets for the treatment of inflammatory diseases (Gao et al., 2021). In the current study, after LPS stimulation, the protein expression levels of iNOS and COX-2 in RAW264.7 cells showed a considerably higher amount than in the control group (Figure 6). The expression of iNOS and COX-2 was significantly down-regulated compared with the LPS group, when **2** has been added at different concentrations (*P* < 0.001). The results indicated that **2** could suppress the NO production by inhibiting the protein expression of iNOS, meanwhile inhibiting protein expression of COX-2 in LPS-induced RAW264.7 cells.

**Figure 7:**
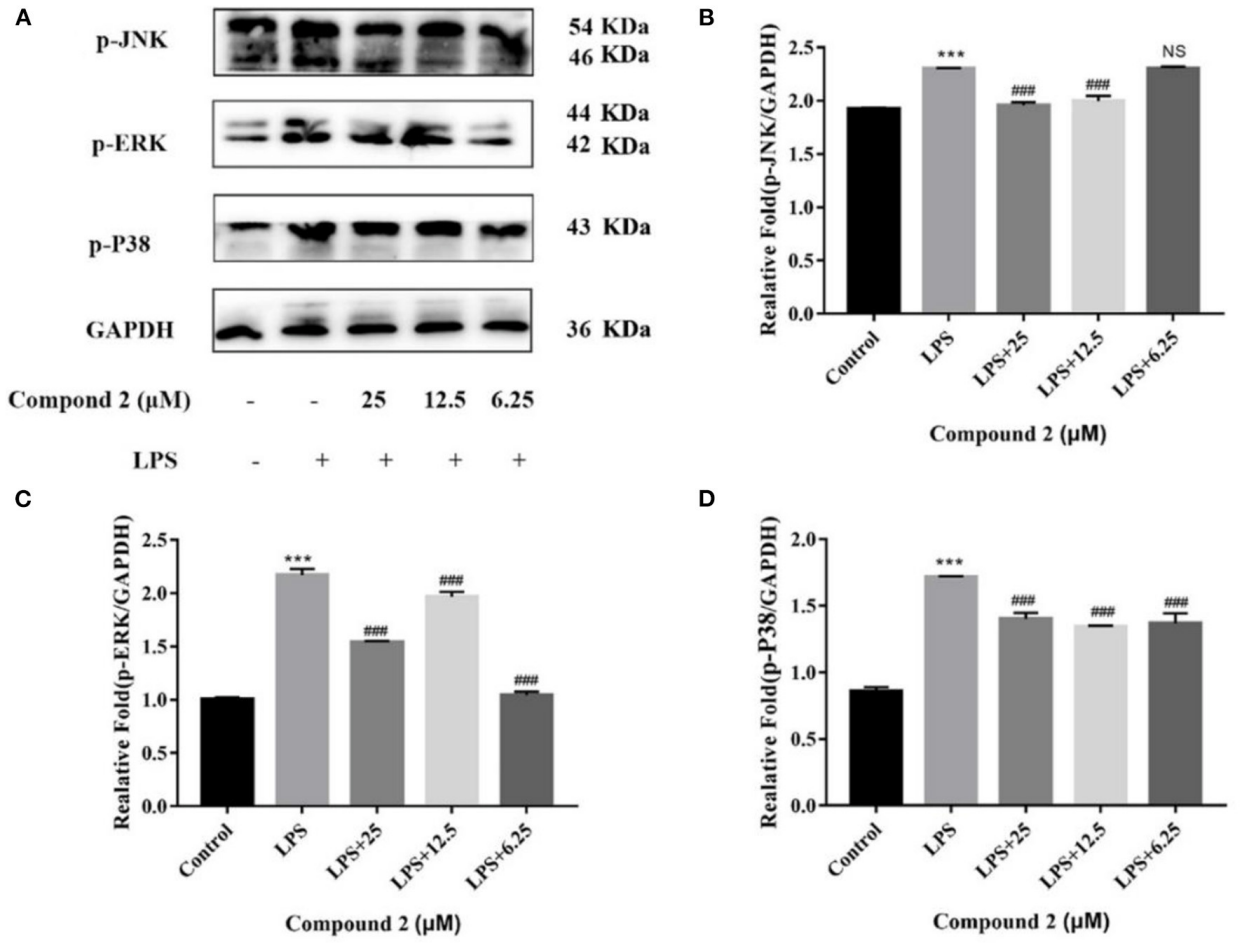
Influences of compound **2** on the MAPK pathway detected by Western blotting. **(A)** The expression levels of p-JNK, p-ERK, p-P38, and GAPDH detected by the western blotting. **(B)** The proportion of p-JNK to GAPDH content. **(C)** The proportion of p-ERK to GAPDH content. **(D)** The proportion of p-P38 to GAPDH content. Data rendered are the mean ± SD, *n* = 3. In comparison to the control, ***P < 0.001. In comparison to the LPS, ^###^*P* < 0.001.

**Figure 8:**
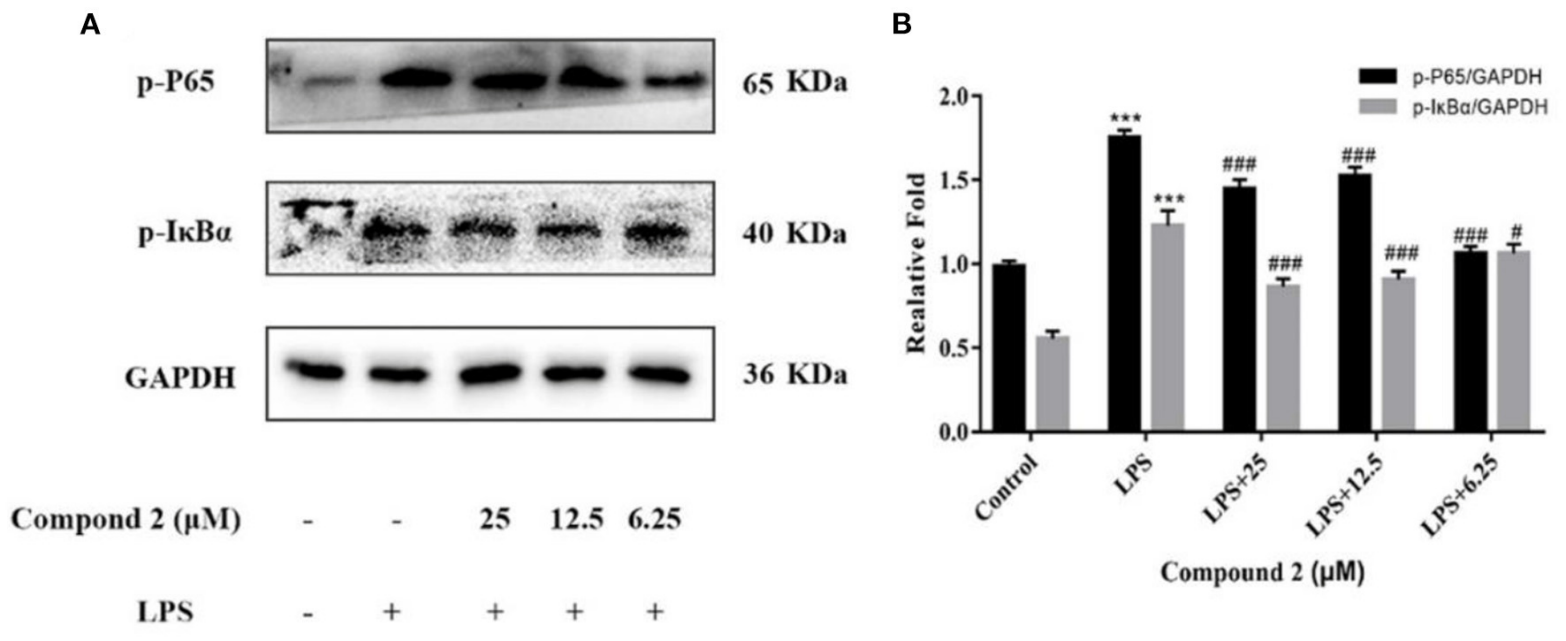
Influences of compound **2** on p-P65, p-IκBα and GAPDH protein expression detected by the western blotting **(A)**. The proportion of p-P65 to GAPDH content and p-IκBα to GAPDH content **(B)**. Data rendered are the mean ± SD, *n* = 3. In comparison to the control, ***P < 0.001. In comparison to the LPS, ^#^*P* < 0.05, ^###^*p* < 0.001.

In macrophages, NF–κB and MAPK signaling pathways were the main signaling pathways controlling inflammatory responses. In NF–κB signaling, key signaling proteins, including IκBα and P65 phosphorylation forms, were chosen as markers of signaling activity; meanwhile, in MAPK signaling pathways, JNK, ERK, and P38 phosphorylation forms were chosen as indicators of signaling activation (Zhang H. et al., 2021). In Figure 7, LPS could significantly upregulate JNK, ERK, and P38 protein phosphorylation in RAW264.7 cells in comparison to the control group (*P* < 0.001). Compound **2** to varying degrees inhibited the expression of JNK, ERK, and P38 proteins phosphorylation in LPS stimulated RAW264.7 cells. In conclusion, the anti-inflammatory function of compound **2** might be connected to the suppressed MAPK signaling pathways in RAW264.7 cells. In Figure 8, LPS remarkably improves the phosphorylation of IκBα and P65 in RAW264.7 cells in comparison to the control group (*P* < 0.001). Compound **2** inhibited the expression of p-P65 and p-IκBα proteins in LPS-induced RAW264.7 cells. In conclusion, the anti-inflammatory effect of compound **2** may be connected to the suppressed NF–κB signaling pathways in RAW264.7 cells.

## Conclusion

In total, five new compounds, including eschscholin B (**2**), dalditone A-B (**3**-**4**), (1*R*, 4*R*)-5-methoxy-1,2,3,4-tetrahydronaphthalene-1,4-dio (**5**), and daldilene A (**6**), were isolated from mangrove endophytic fungus *D. eschscholtzii*. Their structures and absolute configurations were determined by spectroscopy data and ECD calculation. The absolute configuration of **1** was first determined by ECD calculation. Compounds **2** and **6** exhibited potent anti-inflammatory activities with IC_50_ values of 19.3 and 12.9 μM, respectively. Compound **2** belongs to the family of macrocyclic ether, which showed various biological activities. For example, eurysoloids B with immunosuppressive and adipogenesis inhibitory activities (Teng et al., 2021), 12*S*, 13*S*-epoxyobtusa-llene IV with cytotoxic activity (Gutiérrez-Cepeda et al., 2016), durumhemiketalolides A and C with anti-inflammatory activity (Cheng et al., 2009) have reported. In addition, further studies showed that compound **2** might play an anti-inflammatory role by inhibiting the activation of MAPK and NF–κB signaling pathways. This study will contribute to the chemical diversity of polyketide and the discovery of potential anti-inflammatory agents from extreme mangrove-derived fungi.

## Data Availability Statement

The datasets presented in this study can be found in online repositories. The names of the repository/repositories and accession number(s) can be found in the article/Supplementary Material.

## Author Contributions

GW performed the experiments and wrote the article. ZY, SW, and YY participated in the experiments. YC and WK reviewed the article. WK designed and supervised the experiments. All authors have read and agreed to the published version of the article.

## Funding

This work was supported by the Key Project in Science and Technology Agency of Henan Province (212102311029 and 222102310236) and the Key Scientific Research Project in Colleges and the Universities of Henan Province (22B350001).

## Conflict of Interest

The authors declare that the research was conducted in the absence of any commercial or financial relationships that could be construed as a potential conflict of interest.

## Publisher's Note

All claims expressed in this article are solely those of the authors and do not necessarily represent those of their affiliated organizations, or those of the publisher, the editors and the reviewers. Any product that may be evaluated in this article, or claim that may be made by its manufacturer, is not guaranteed or endorsed by the publisher.
