# Supplementary material for: Diversified Polyketides With Anti-inflammatory Activities From Mangrove Endophytic Fungus Daldinia eschscholtzii KBJYZ-1
Source: Front Microbiol. 2022 May 10;13:900227. doi: 10.3389/fmicb.2022.900227 (PMC9127611; doi:10.3389/fmicb.2022.900227)
Supplement: Supplementary file 1 [file Table_1.DOCX]

Supplementary Material

Diversified Polyketides with Anti-inflammatory Activities from Mangrove Endophytic Fungus *Daldinia eschscholtzii* KBJYZ-1

**Guisheng Wang^1^, Zhenhua Yin^1^, Senye Wang^1^, Yilin Yuan^1^, Yan Chen^1,2*^, Wenyi Kang^1,2,3*^**

^1^National R & D Center for Edible Fungus Processing Technology, Henan University, Kaifeng 475004, China;

^2^Joint International Research Laboratory of Food & Medicine Resource Function, Henan Province, Kaifeng 475004, China;

^3^Kaifeng Key Laboratory of Functional Components in Health Food, Kaifeng 475004, China.

***Correspondence:**

Yan Chen: [cychemistry@163.com](mailto:cychemistry@163.com)

Wenyi Kang: [kangwenyi@henu.edu.cn](mailto:kangwenyi@henu.edu.cn)

Figure S1. HRESIMS spectrum of eschscholin B (**2**)

Figure S2. ^1^H-NMR spectrum of eschscholin B (**2**) in CDCl_3_

Figure S3. ^13^C-NMR spectrum of eschscholin B (**2**) in CDCl_3_

Figure S4. ^1^H-^1^H COSY spectrum of eschscholin B (**2**) in CDCl_3_

Figure S5. HSQC spectrum of eschscholin B (**2**) in CDCl_3_

Figure S6. HMBC spectrum of eschscholin B (**2**) in CDCl_3_

Figure S7. NOESY spectrum of eschscholin B (**2**) in CDCl_3_

Figure S8. HRESIMS spectrum of dalditone A (**3**)

Figure S9. ^1^H-NMR spectrum of dalditone A (**3**) in MeOH-*d*_4_

Figure S10. ^13^C-NMR spectrum of dalditone A (**3**) in MeOH-*d*_4_

Figure S11. ^1^H-^1^H COSY spectrum of dalditone A (**3**) in MeOH-*d*_4_

Figure S12. HSQC spectrum of dalditone A (**3**) in MeOH-*d*_4_

Figure S13. HMBC spectrum of dalditone A (**3**) in MeOH-*d*_4_

Figure S14. HRESIMS spectrum of dalditone B (**4**)

Figure S15. ^1^H-NMR spectrum of dalditone B (**4**) in MeOH-*d*_4_

Figure S16. ^13^C-NMR spectrum of dalditone B (**4**) in MeOH-*d*_4_

Figure S17. ^1^H-^1^H COSY spectrum of dalditone B (**4**) in MeOH-*d*_4_

Figure S18. HSQC spectrum of dalditone B (**4**) in MeOH-*d*_4_

Figure S19. HMBC spectrum of dalditone B (**4**) in MeOH-*d*_4_

Figure S20. HRESIMS spectrum of (1*R*,4*R*)-5-methoxy-1,2,3,4-tet-rahydronaphtha -lene-1,4-dio (**5**)

Figure S21. ^1^H-NMR spectrum of (1*R*,4*R*)-5-methoxy-1,2,3,4-tetrahydronaphthalene-1,4-dio (**5**) in

CDCl_3_

Figure S22. ^13^C-NMR spectrum of (1*R*,4*R*)-5-methoxy-1,2,3,4-tetrahydronaphthalene-1,4-dio (**5**) in CDCl_3_

Figure S23. ^1^H-^1^H COSY spectrum of (1*R*,4*R*)-5-methoxy-1,2,3,4- tetrahydronaphthal-ene-1,4-dio (**5**) in CDCl_3_

Figure S24. HSQC spectrum of (1*R*,4*R*)-5-methoxy-1,2,3,4-tetrahydronaphthalene-1,4-dio (**5**) in CDCl_3_

Figure S25. HMBC spectrum of (1*R*,4*R*)-5-methoxy-1,2,3,4-tetrahydronaphthalene-1,4-dio (**5**) in CDCl_3_

Figure S26. NOESY spectrum of (1*R*,4*R*)-5-methoxy-1,2,3,4-tetrahydronaphthalene-1,4-dio (**5**) in CDCl_3_

Figure S27. HRESIMS spectrum of daldilene A (**6**)

Figure S28. ^1^H-NMR spectrum of daldilene A (**6**) in CDCl_3_

Figure S29. ^13^C-NMR spectrum of daldilene A (**6**) in CDCl_3_

Figure S30. ^1^H-^1^H COSY spectrum of daldilene A (**6**) in CDCl_3_

Figure S31. HSQC spectrum of daldilene A (**6**) in CDCl_3_

Figure S32. HMBC spectrum of daldilene A (**6**) in CDCl_3_

Table S1. ^1^H and ^13^C NMR data of eschscholin A (**1**) in CDCl_3_.

Figure S33. Optimized low-energy conformers of 12*S*-**1** at the B3LYP/6-31G (d) level.

Table S2. Energy analysis for the conformers of compound **1**.

Table S3. Cartesian coordinates for the low-energy optimized conformers of **1** at B3LYP/DGDZVP level of theory in CH_3_OH.

Figure S34. Optimized low-energy conformers of 12*R*,13*S*-**2** at the B3LYP/6-31G (d) level.

Table S4. Energy analysis for the conformers of compound **2**.

Table S5. Cartesian coordinates for the low-energy optimized conformers of **2** at PBEPBE/6-311+G level of theory in CH_3_OH.

Figure S35. Optimized low-energy conformers of 12*S*-**4** at the B3LYP/6-31G (d) level.

Table S6. Energy analysis for the conformers of compound **4**.

Table S7. Cartesian coordinates for the low-energy optimized conformers of **4** at B3LYP/6-31G level of theory in CH_3_OH.

Figure S36. Optimized low-energy conformers of 1*S*,4*S*-**5** at the B3LYP/6-31G (d) level.

Table S8. Energy analysis for the conformers of compound **5**.

Table S9. Cartesian coordinates for the low-energy optimized conformers of **5** at B3LYP/6-311G level of theory in CH_3_OH.

Figure S37. The IR spectrum of **2**.

Figure S38. The UV spectrum of **2**.

Figure S39. The IR spectrum of **6**.

Figure S40. The UV spectrum of **6**.

Figure S1. HRESIMS spectrum of compound **2**


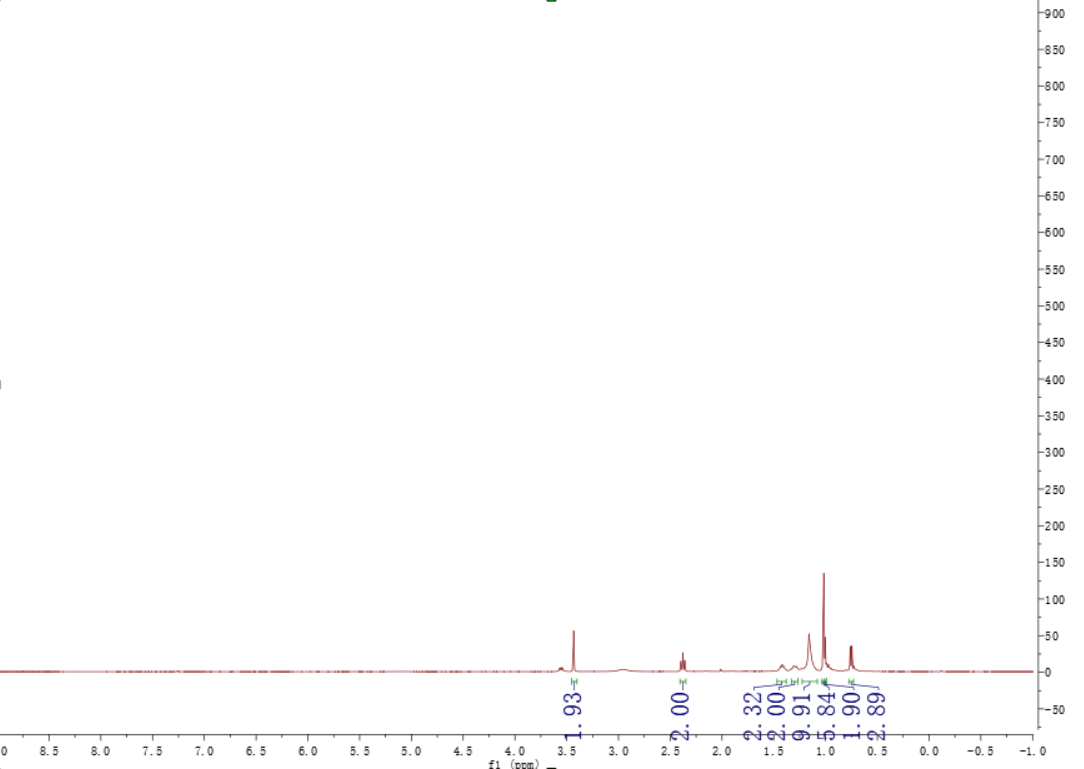


Figure S2. ^1^H NMR spectrum of compound **2** (500 MHz, CDCl_3_)


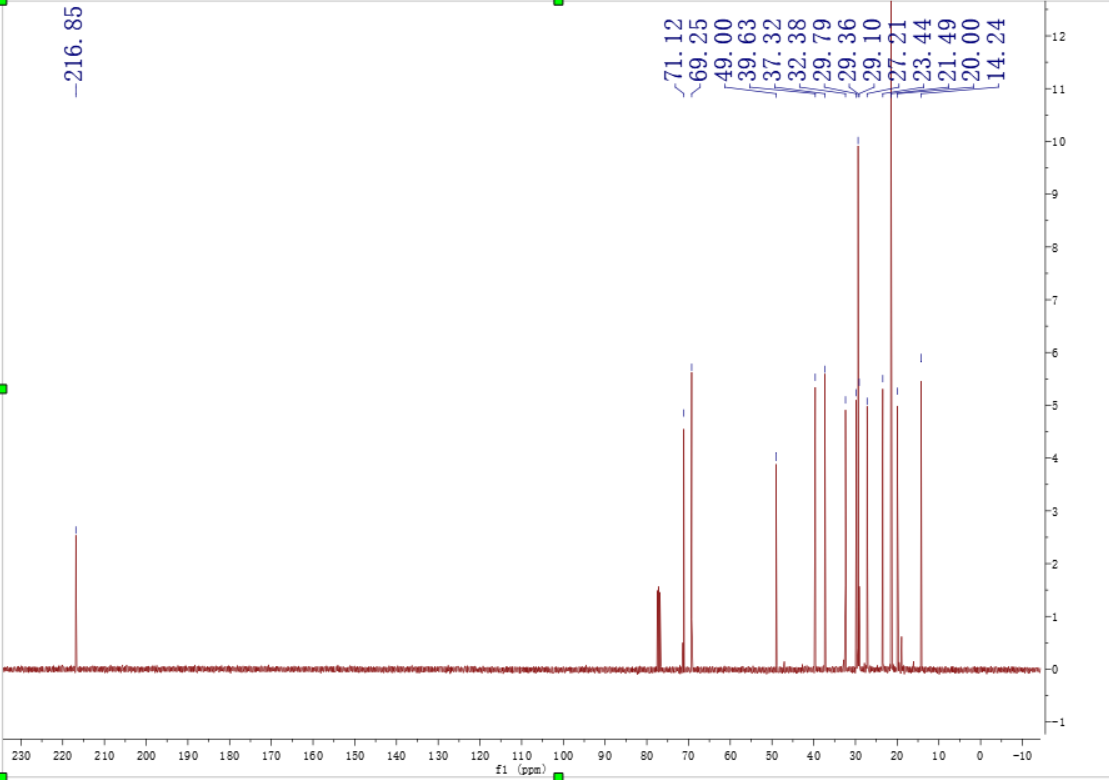


Figure S3. ^13^C NMR spectrum of compound **2** (125 MHz, CDCl_3_)


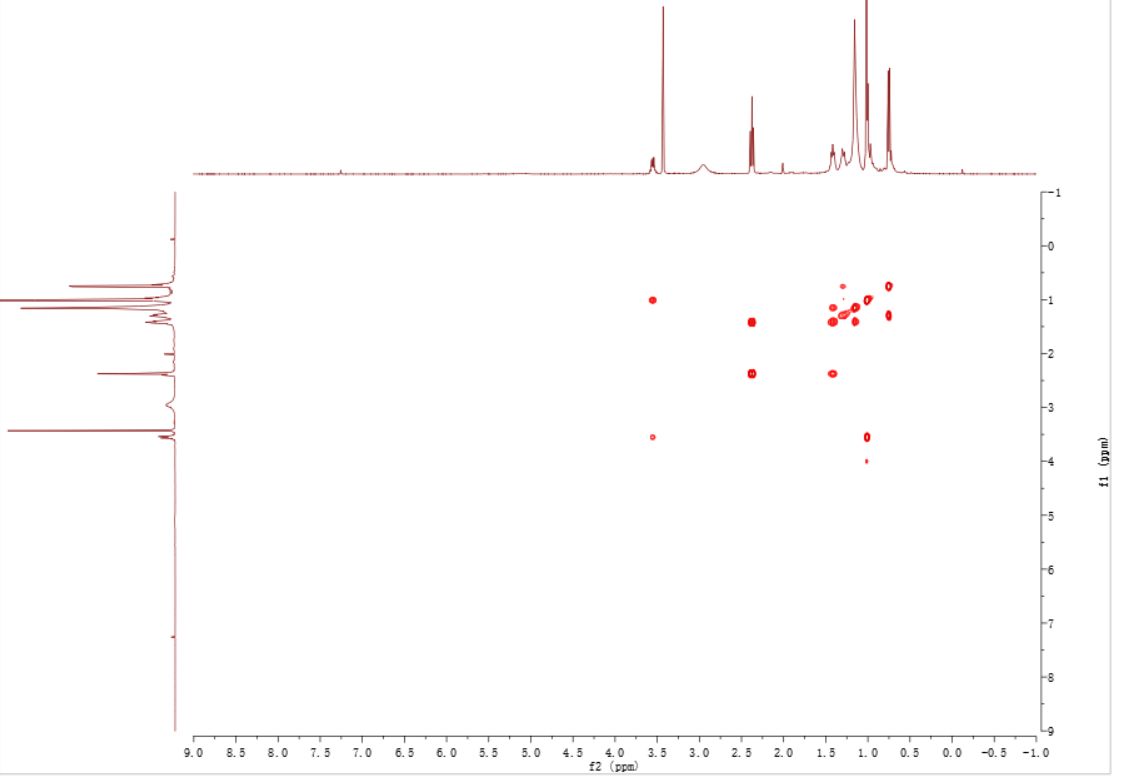


Figure S4. ^1^H-^1^H COSY spectrum of compound **2**


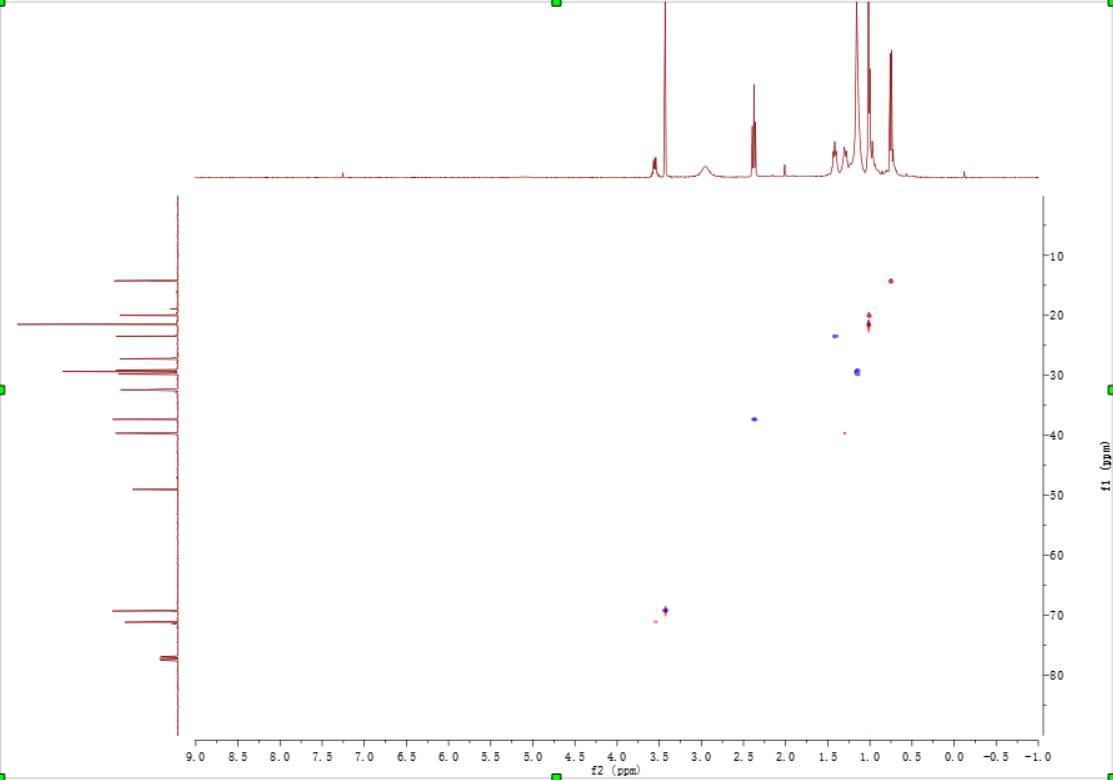


Figure S5. HSQC spectrum of compound **2**


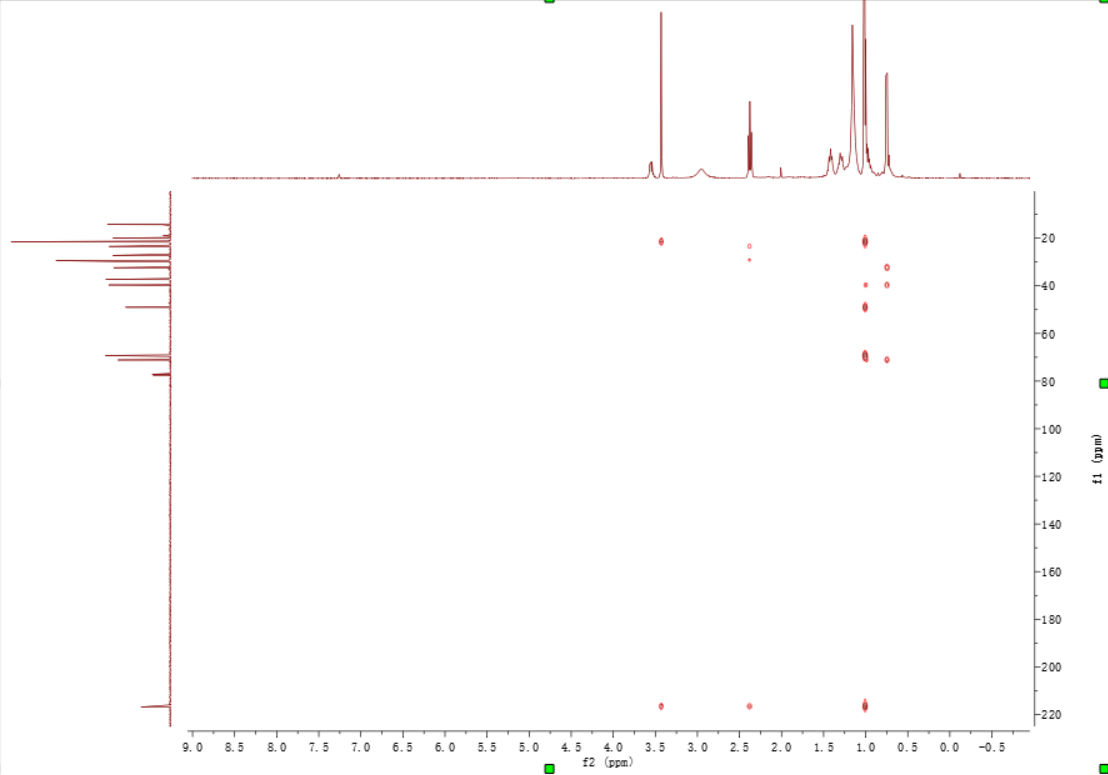


Figure S6. HMBC spectrum of compound **2**


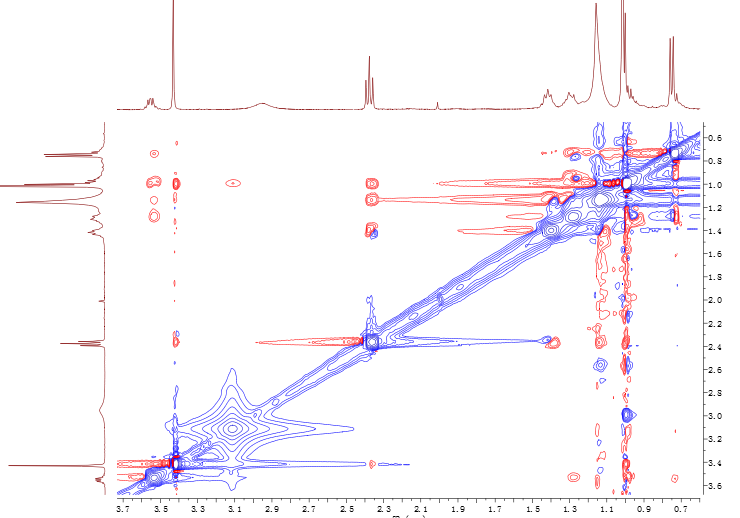


Figure S7. NOESY spectrum of compound **2**


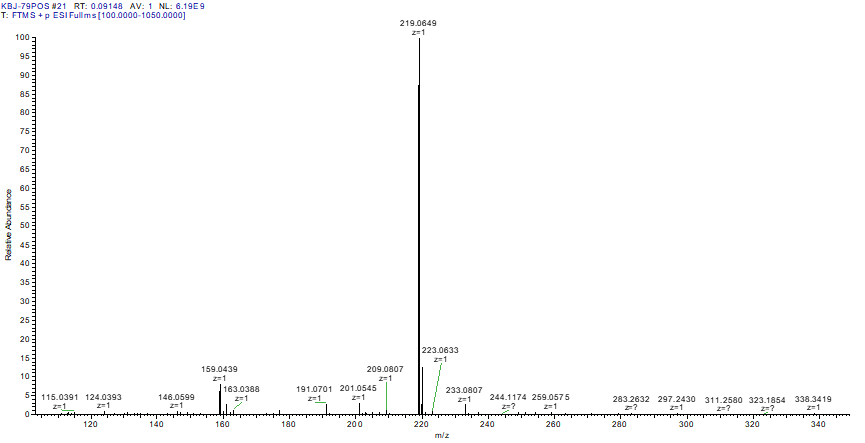


Figure S8. HRESIMS spectrum of compound **3**


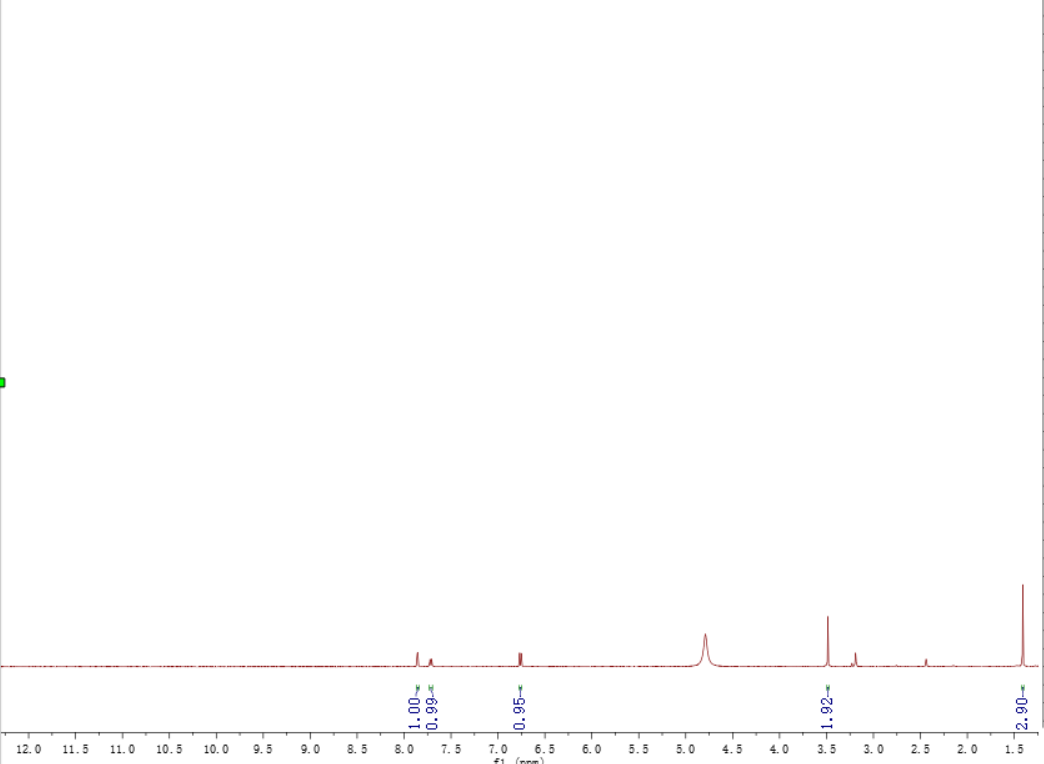


Figure S9. ^1^H NMR spectrum of compound **3** (500 MHz, MeOH-*d*_4_)


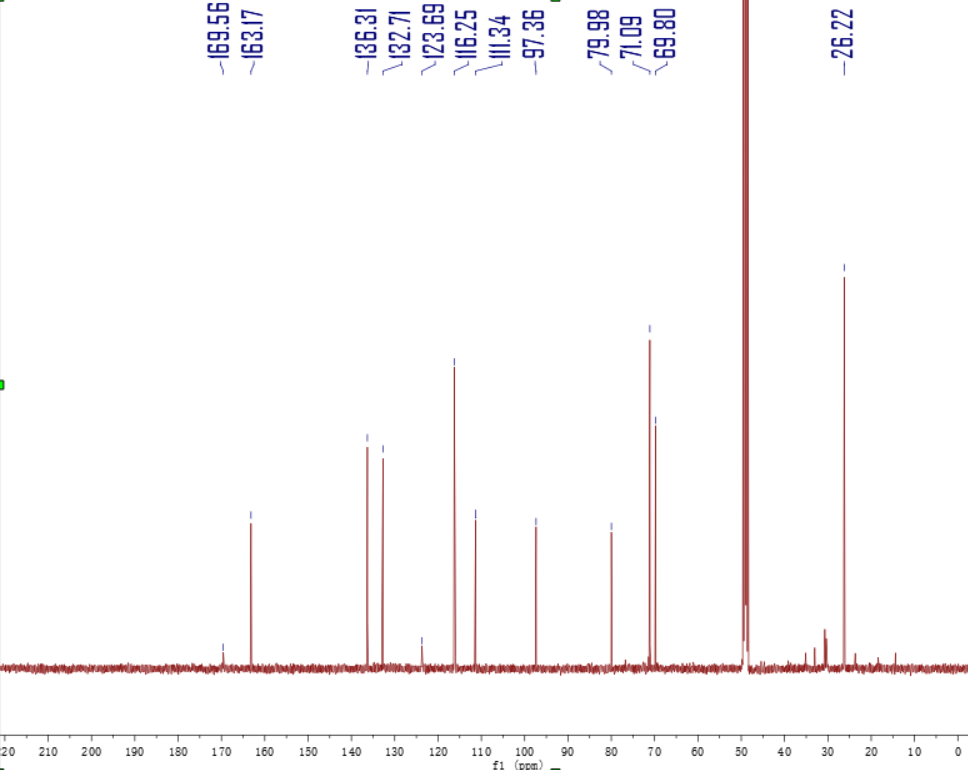


Figure S10. ^13^C NMR spectrum of compound **3** (500 MHz, MeOH-*d*_4_)


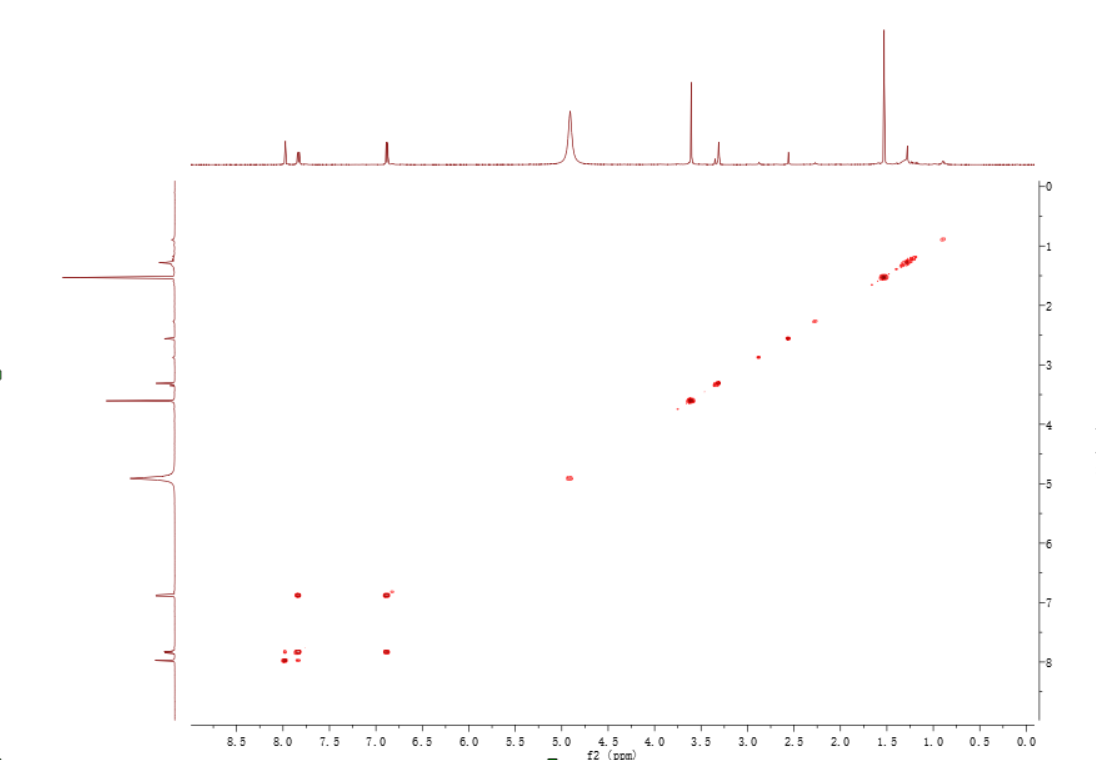


Figure S11. ^1^H-^1^H COSY spectrum of compound **3**


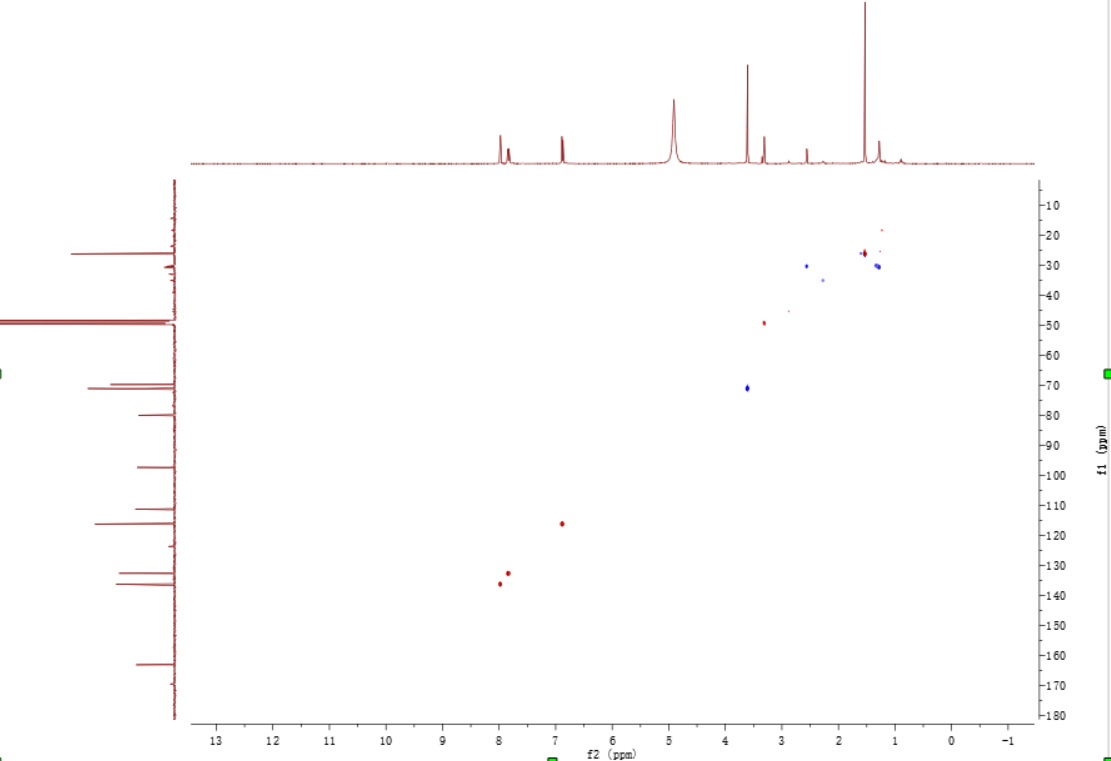


Figure S12. HSQC spectrum of compound **3**


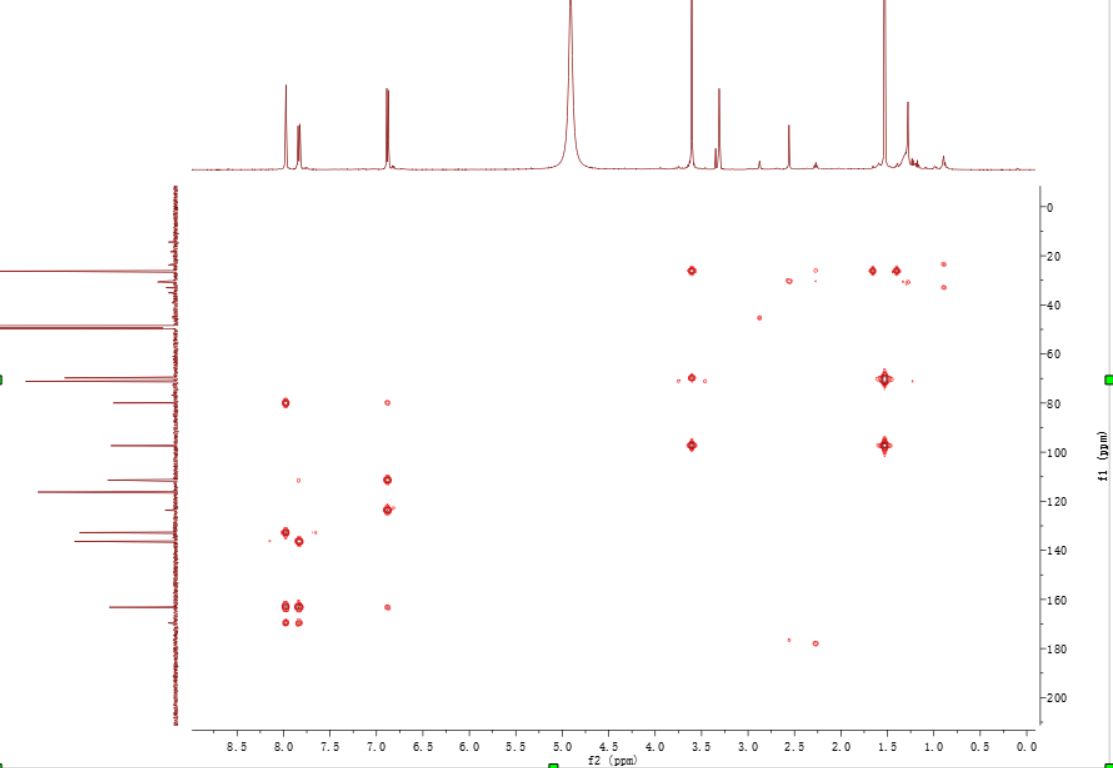


Figure S13. HMBC spectrum of compound **3**

Figure S14. HRESIMS spectrum of compound **4**


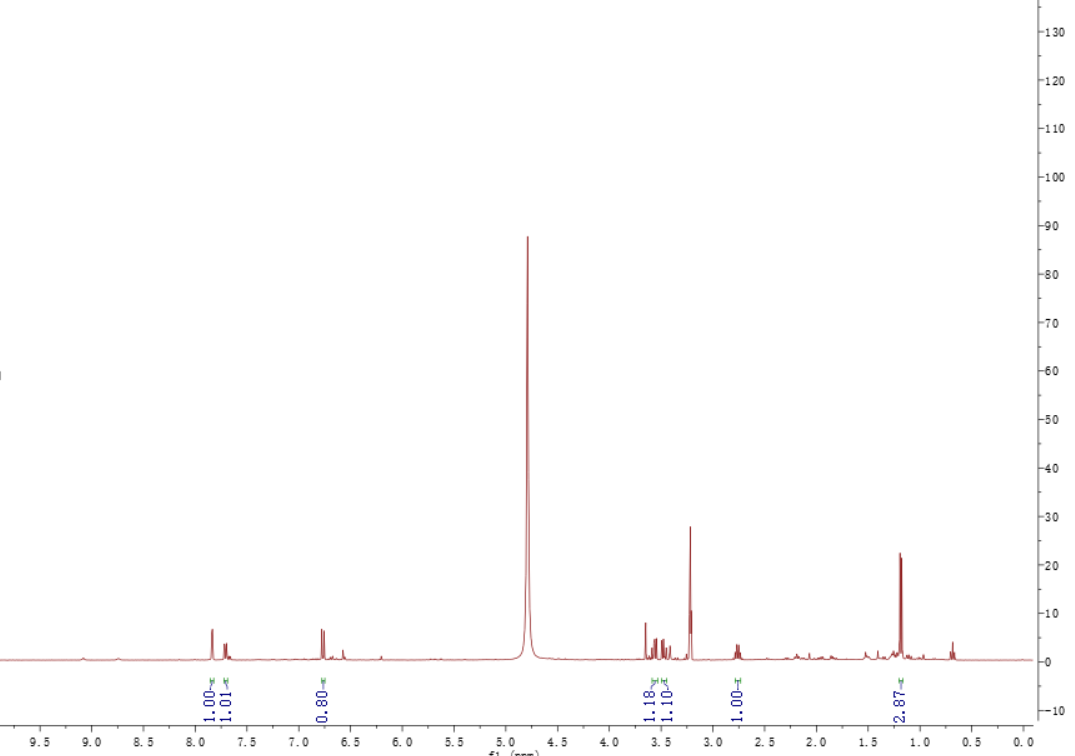


Figure S15. ^1^H NMR spectrum of compound **4** (500 MHz, MeOH-*d*_4_)


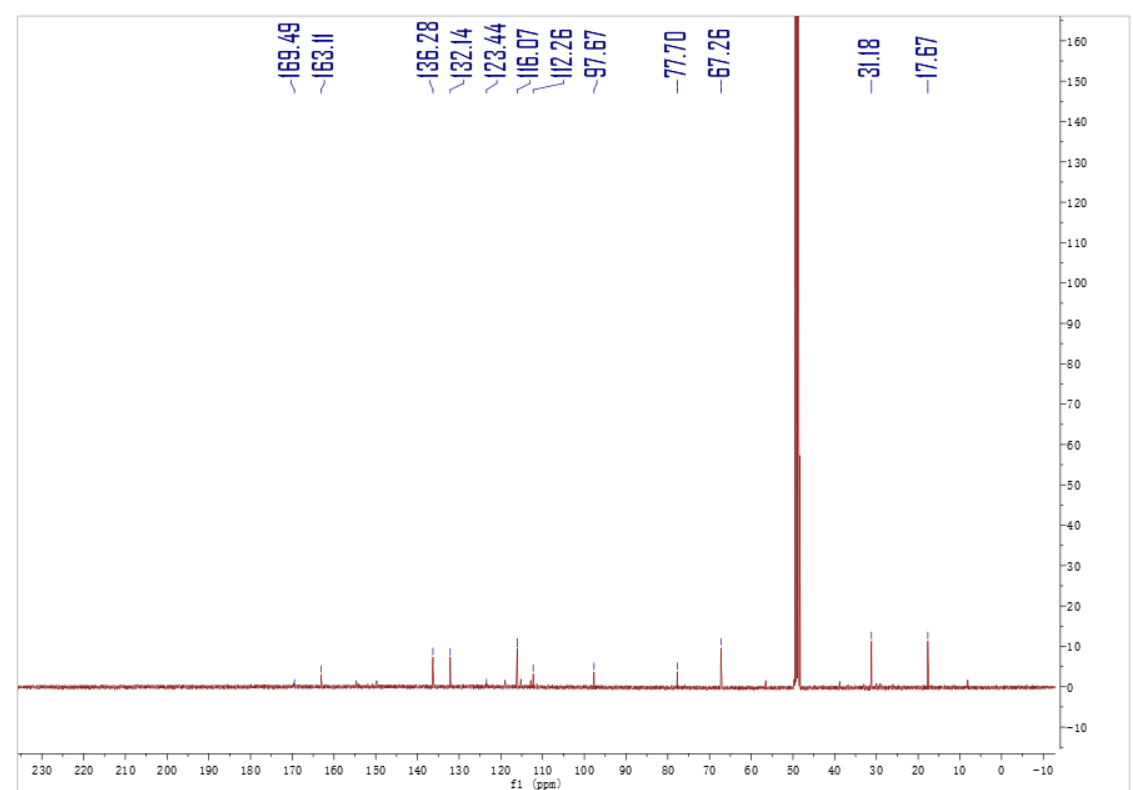


Figure S16. ^13^C NMR spectrum of compound **4** (125 MHz, MeOH-*d*_4_).


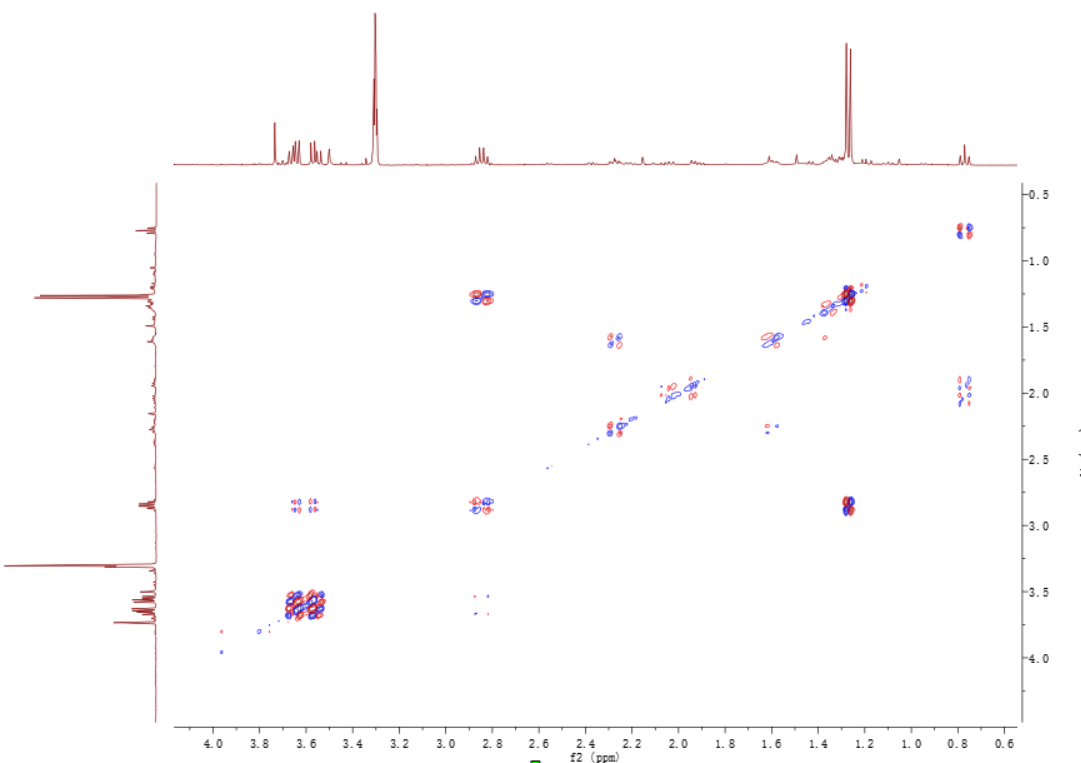


Figure S17. ^1^H-^1^H COSY spectrum of compound **4**


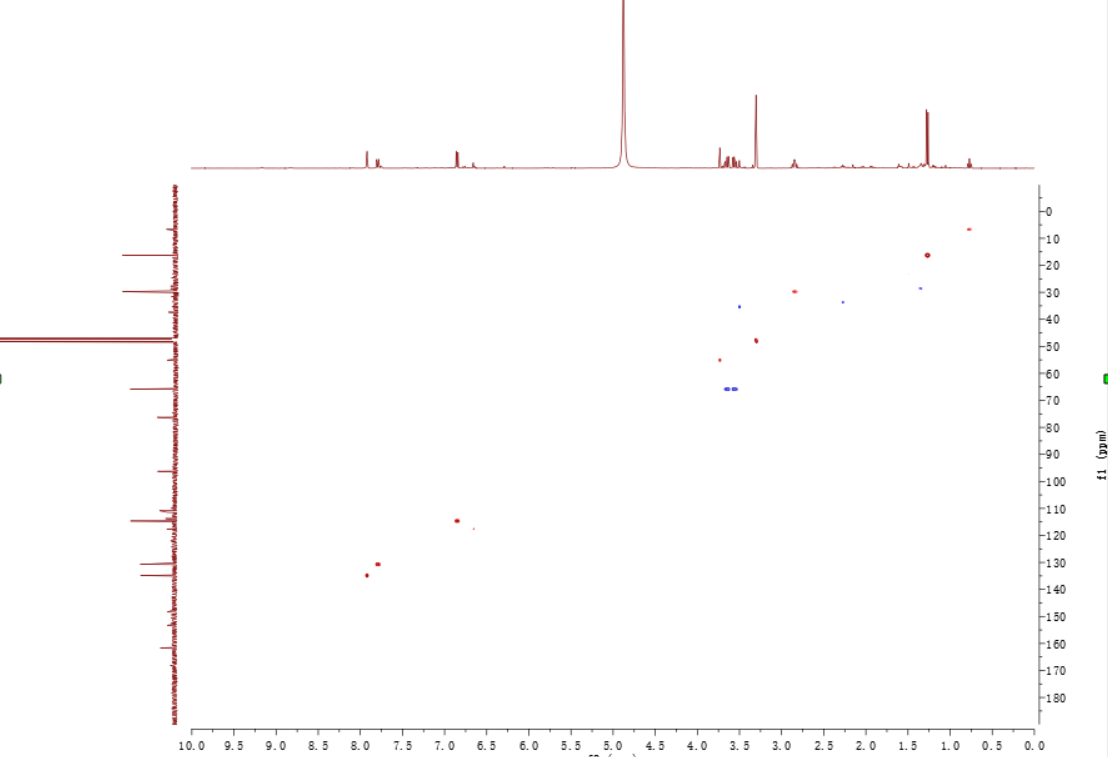


Figure S18. HSQC spectrum of compound **4**


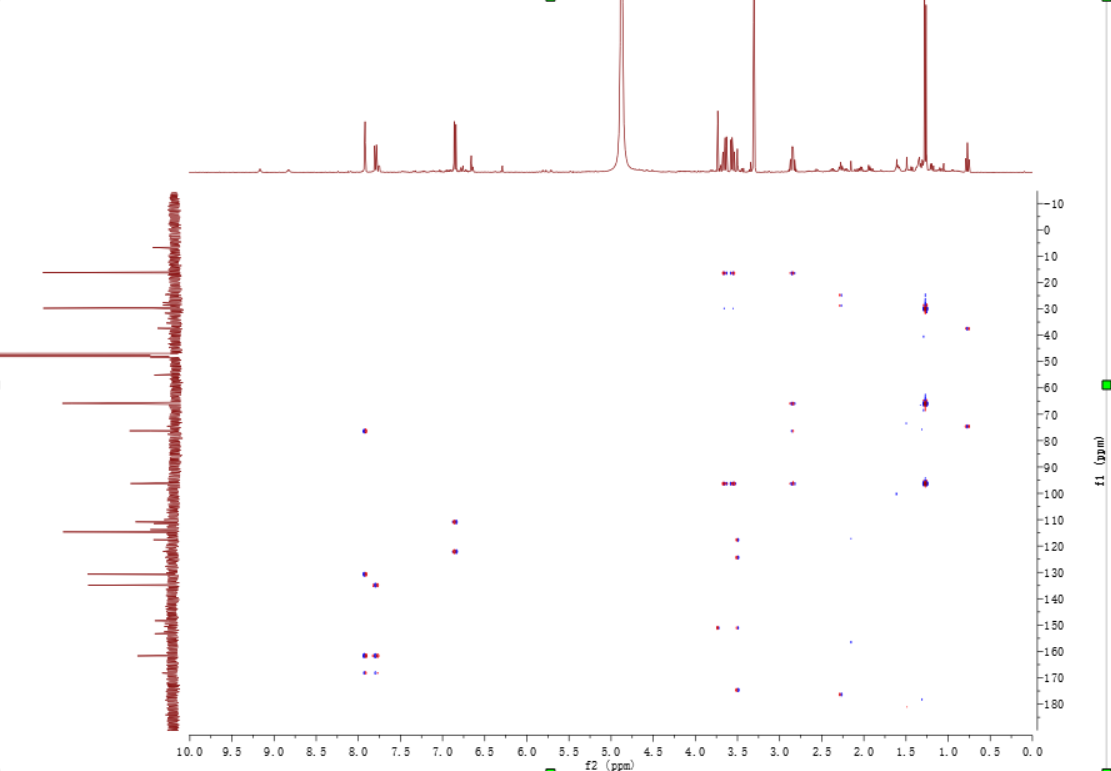


Figure S19. HMBC spectrum of compound **4**.


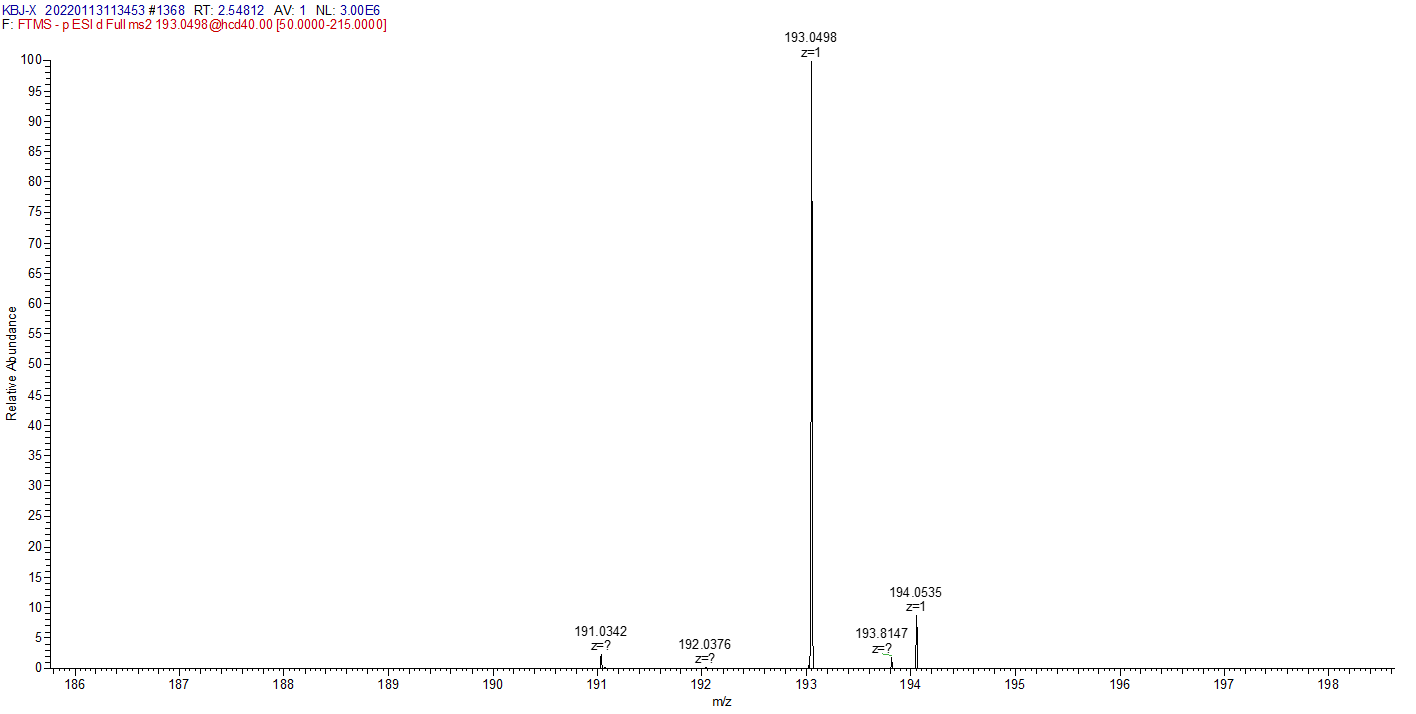


Figure S20. HRESIMS spectrum of compound **5**


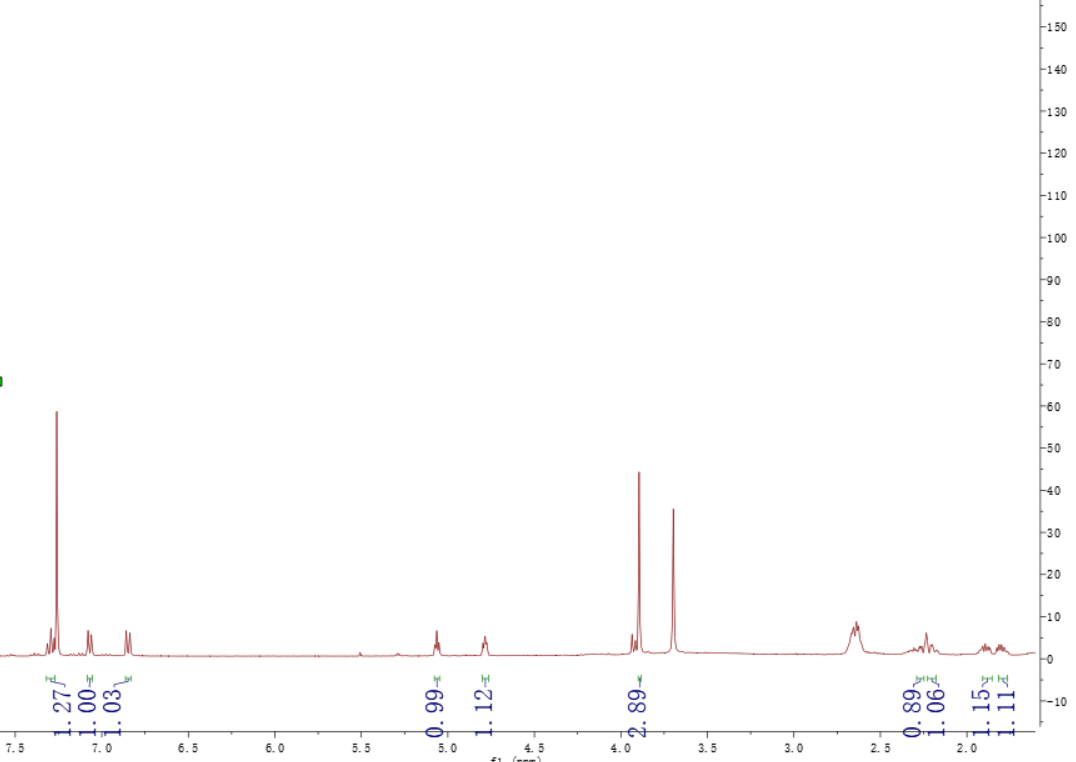


Figure S21. ^1^H NMR spectrum of compound **5** (500 MHz, CDCl_3_)


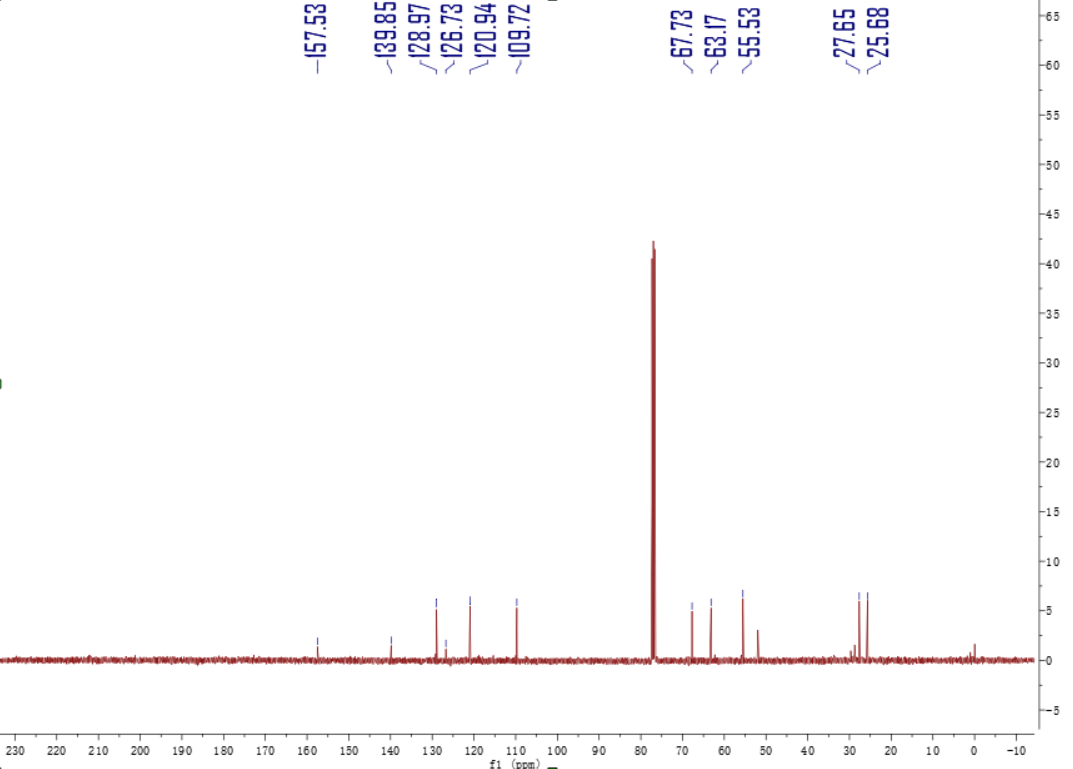


Figure S22. ^13^C NMR spectrum of compound **5** (500 MHz, CDCl_3_)


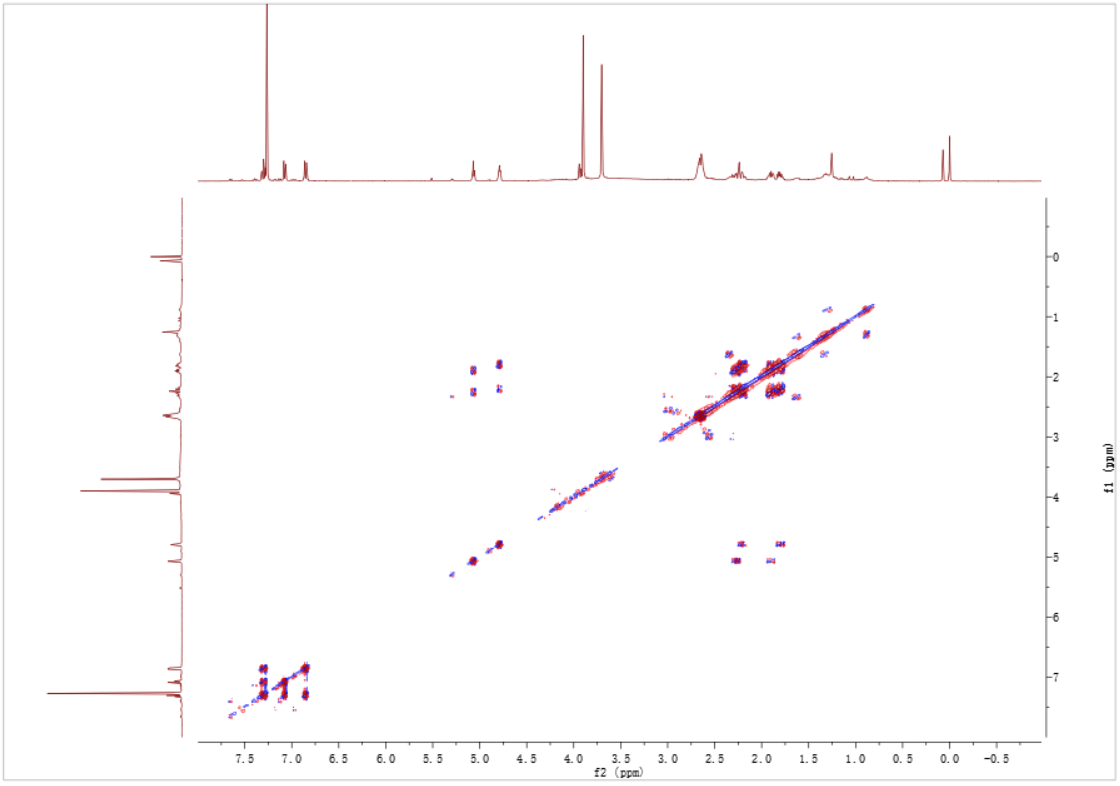


Figure S23. ^1^H-^1^H COSY spectrum of compound **5**


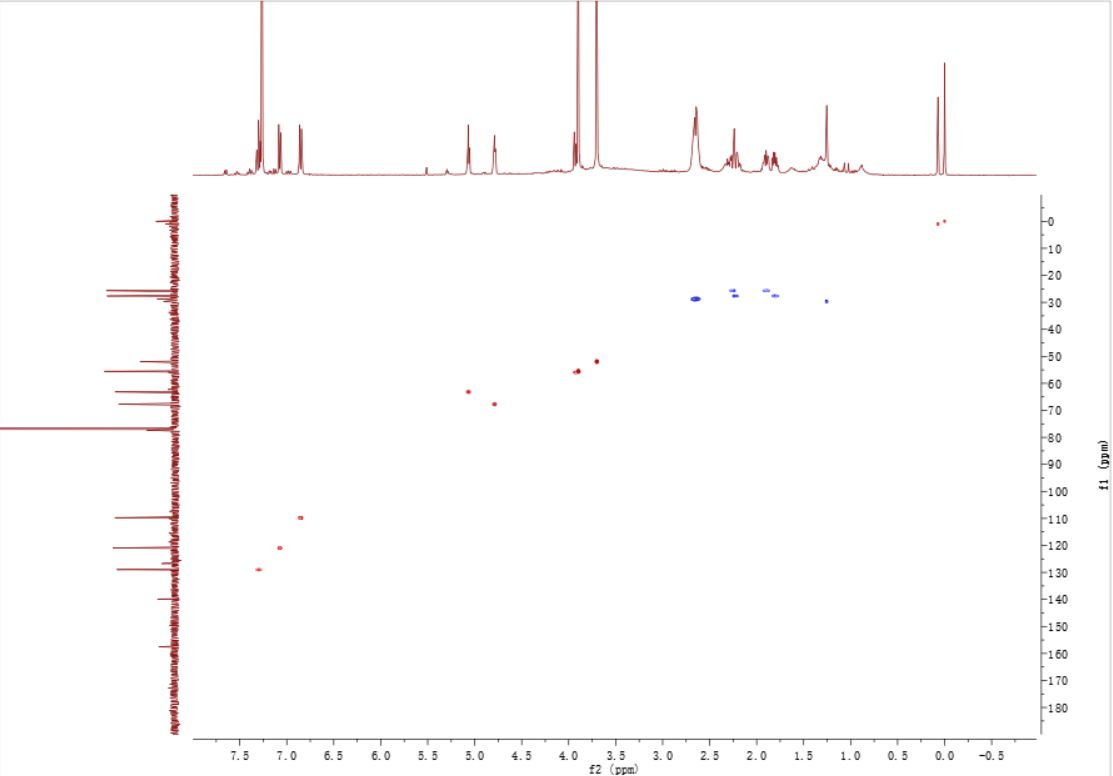


Figure S24. HSQC spectrum of compound **5**


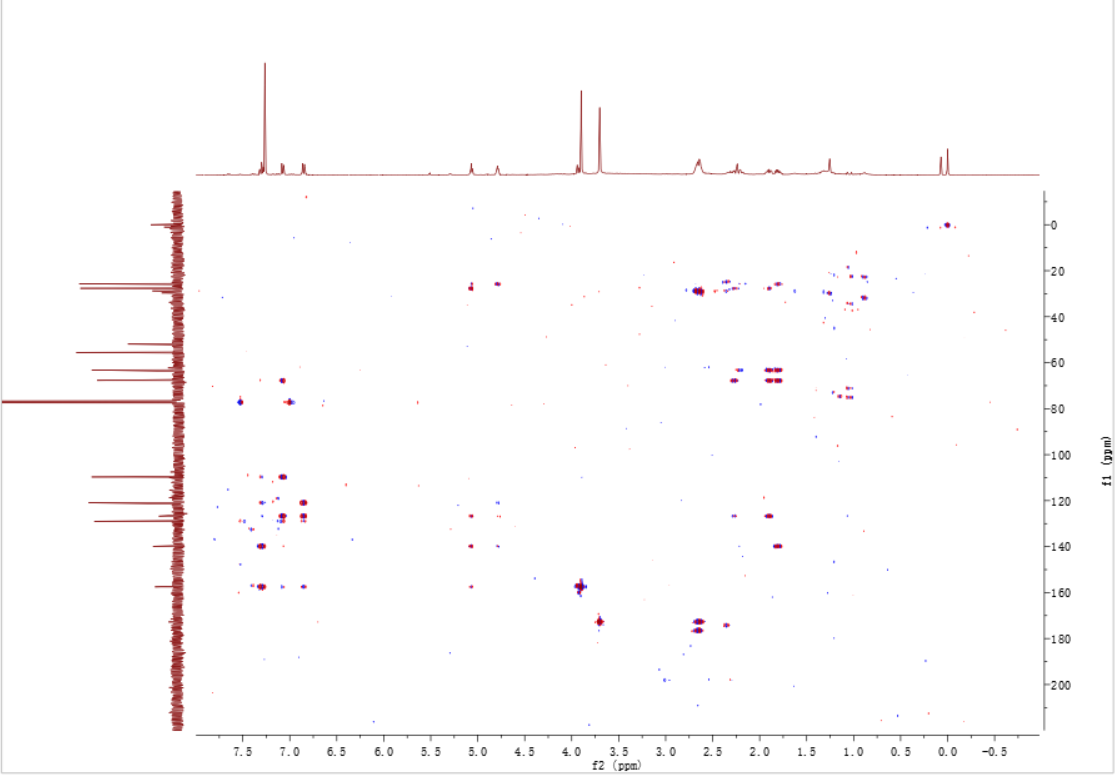


Figure S25. HMBC spectrum of compound **5**


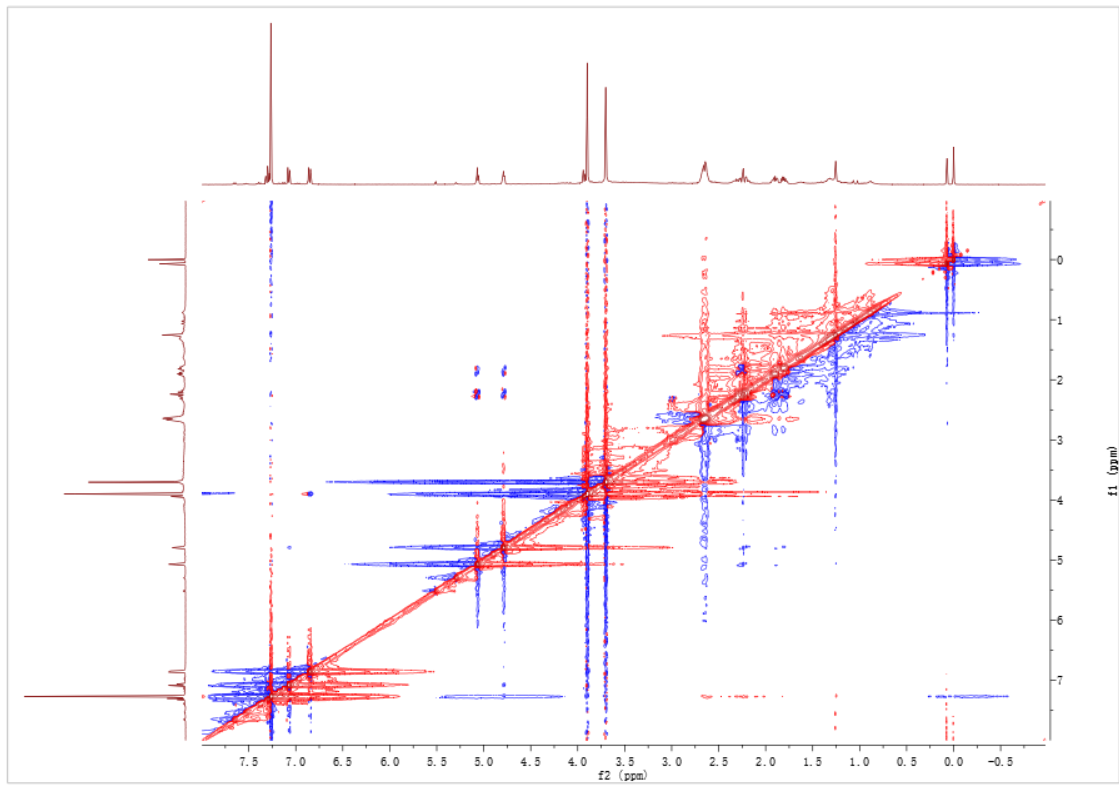


Figure S26. NOESY spectrum of compound **5**

Figure S27. HRESIMS spectrum of compound **6**


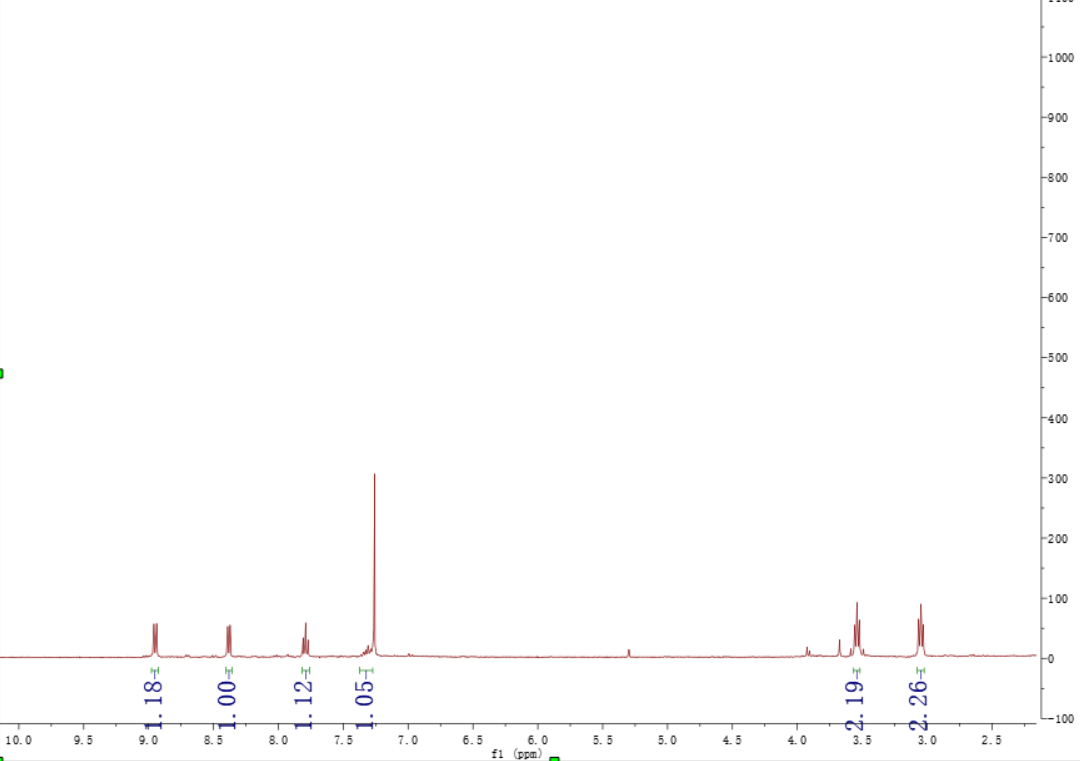


Figure S28. ^1^H NMR spectrum of compound **6** (500 MHz, CDCl_3_)


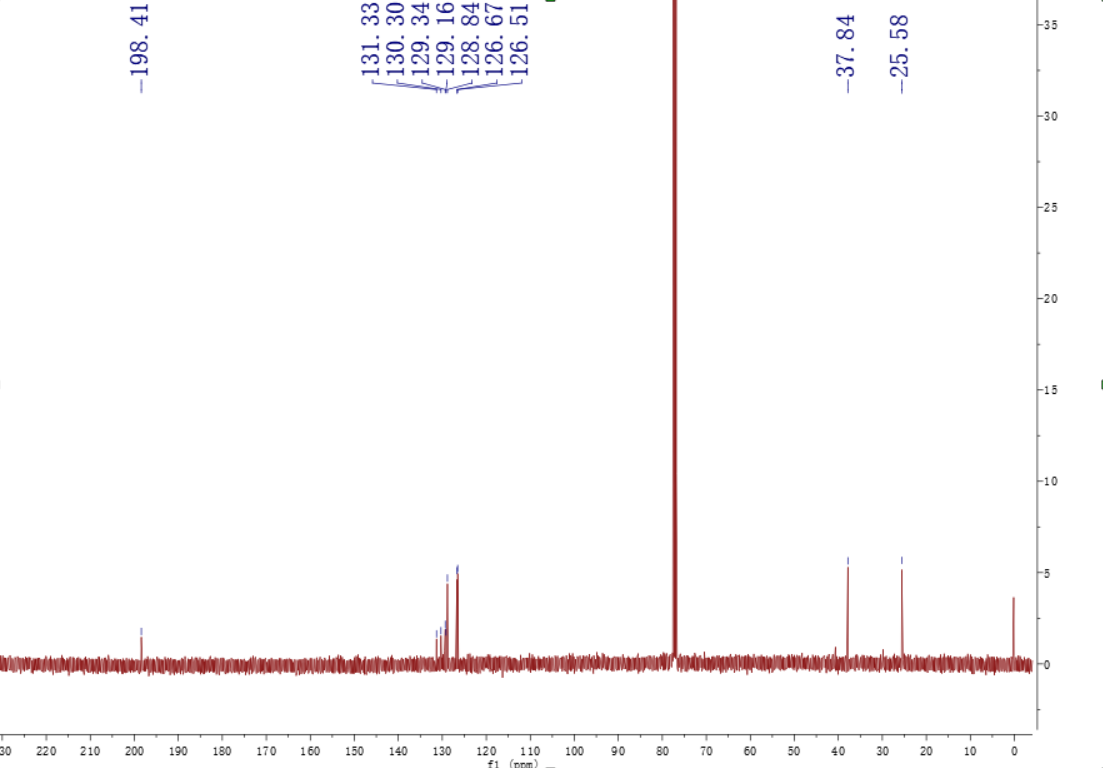


Figure S29. ^13^C NMR spectrum of compound **6** (125 MHz, CDCl_3_)


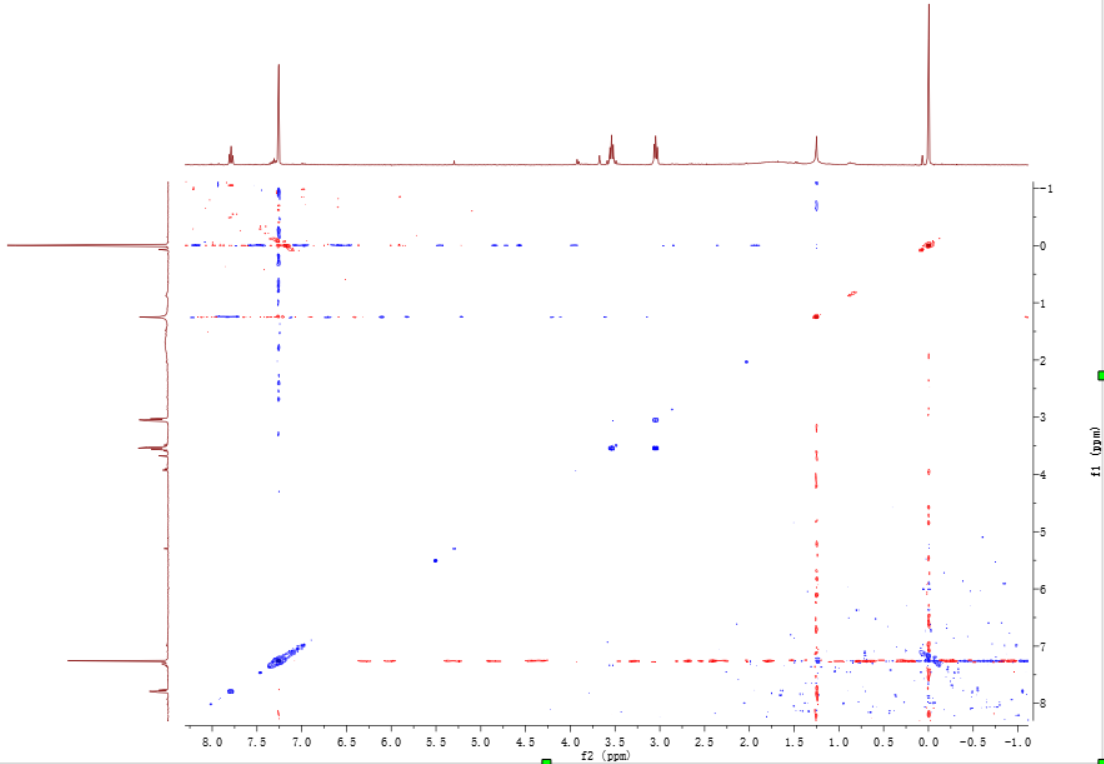


Figure S30. ^1^H-^1^H COSY spectrum of compound **6**


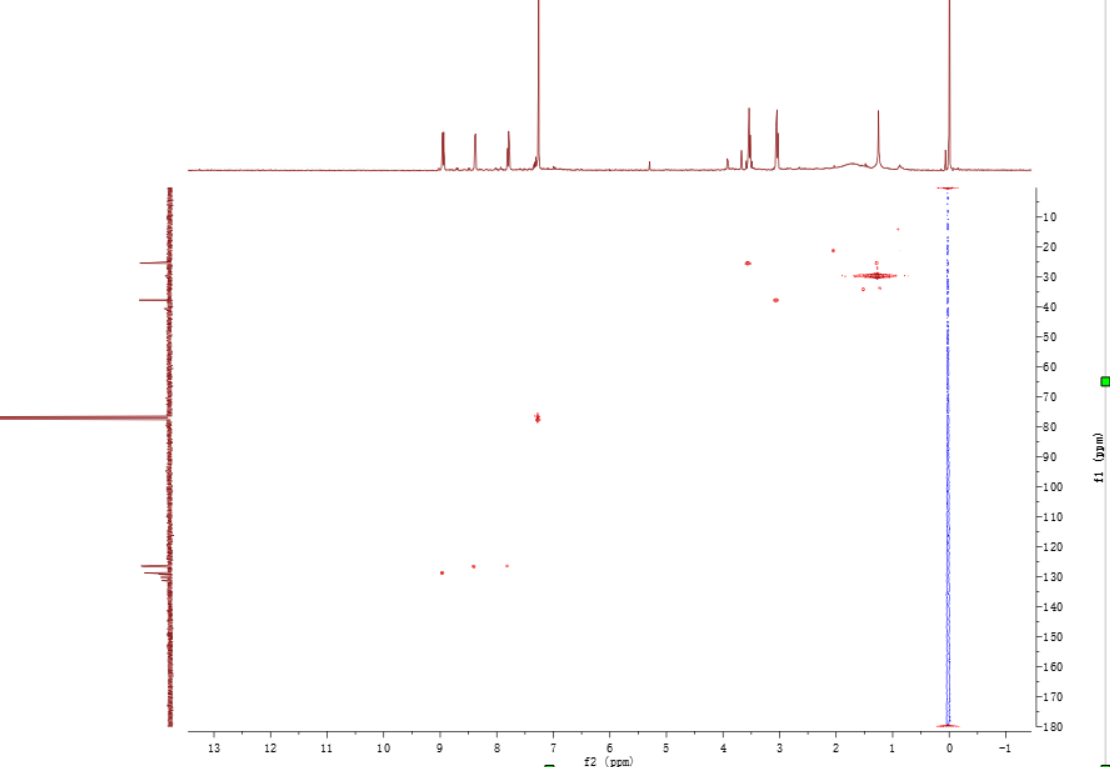


Figure S31. HSQC spectrum of compound **6**


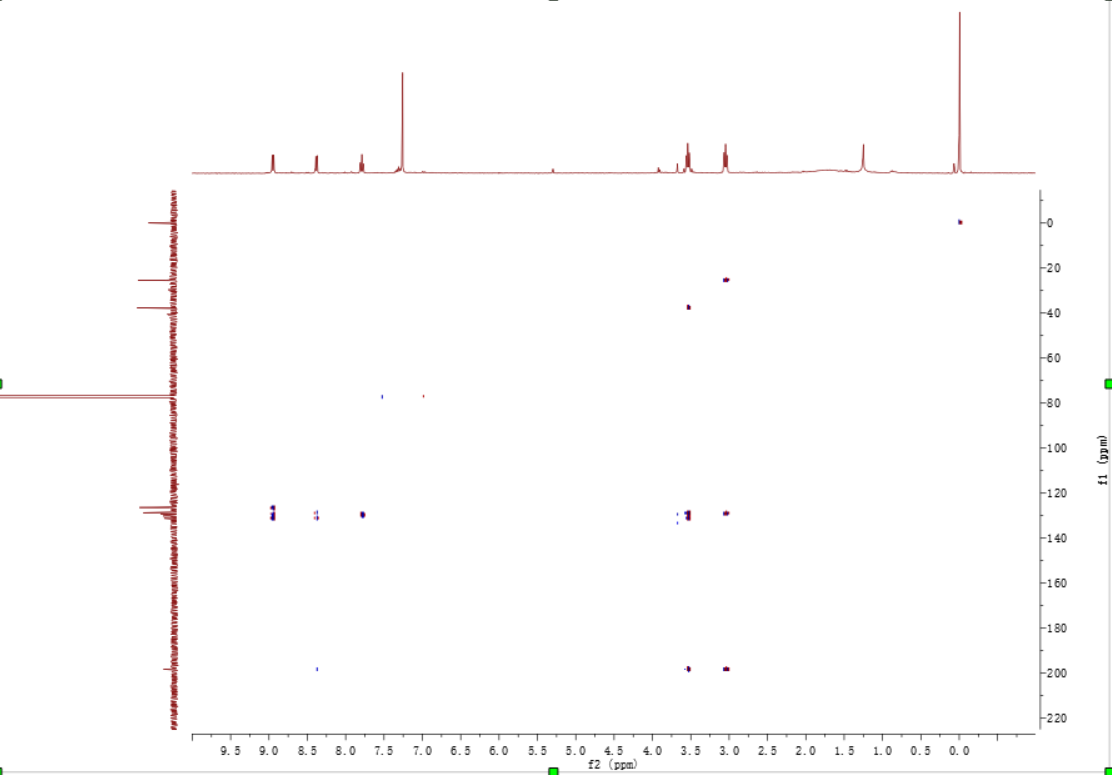


Figure S32. HMBC spectrum of compound **6**

Table S1. ^1^H and ^13^C NMR data of eschscholin A (**1**) in CDCl_3_.

| no. | *δ*_C_ | *δ*_H_ (mult, *J* (Hz)) | no. | *δ*_C_ | *δ*_H_ (mult, *J* (Hz)) |
| --- | --- | --- | --- | --- | --- |
| 1 | 64.6, CH_2_ | 3.55, s | 10 | 27.2, CH_2_ | 1.22, overlap |
| 2 | 49.0, C |  | 11a | 32.9, CH_2_ | 1.64, m |
| 3 | 217.3, C |  | 11b |  | 1.34, m |
| 4 | 37.3, CH_2_ | 2.48, m | 12 | 39.6, CH | 2.48, m |
| 5 | 23.5, CH_2_ | 1.54, m | 13 | 71.1, CH |  |
| 6 | 29.2, CH_2_ | 1.27, overlap | 14 | 14.2, CH3 | 2.13, s |
| 7 | 29.4, CH_2_ | 1.27, overlap | 15 | 20.0, CH3 | 1.08, d (6.8) |
| 8 | 29.3, CH_2_ | 1.27, overlap | 16 | 21.5, CH3 | 1.15, s |
| 9 | 29.6, CH_2_ | 1.27, overlap | 17 | 21.5, CH3 | 1.15, s |


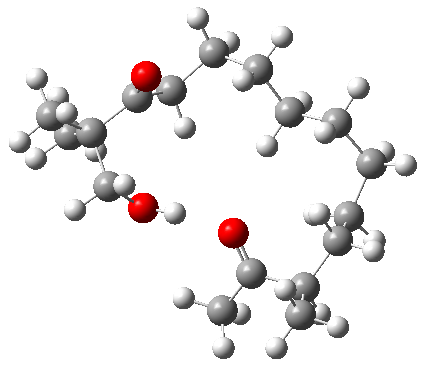

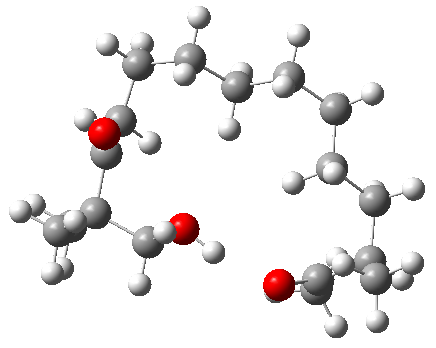

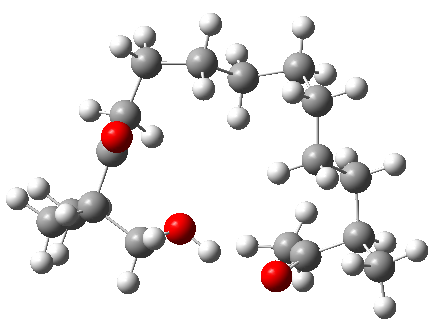


**1a 1b 1c**


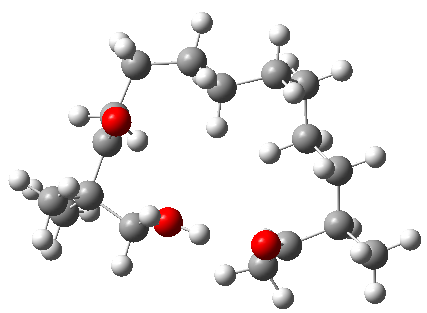

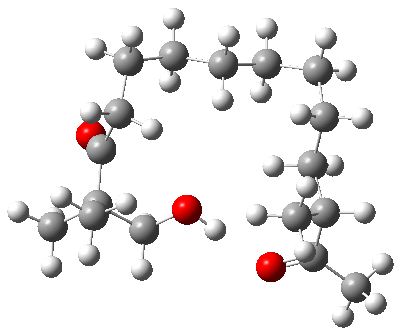

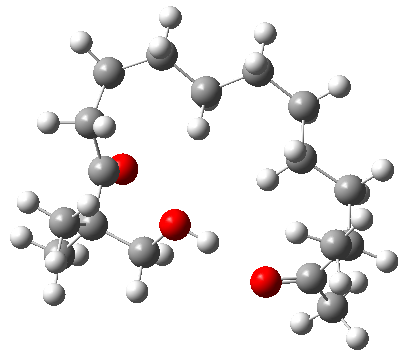


**1d 1e 1f**


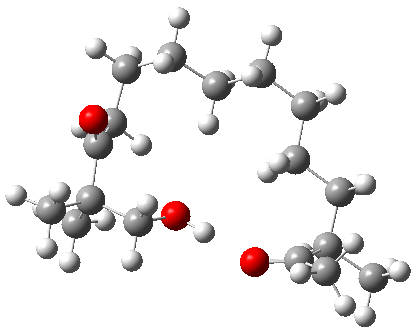

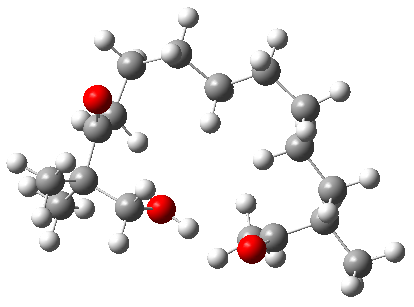


**1g 1h**

**Figure S33.** Optimized low-energy conformers of 12*S*-**1** at the B3LYP/6-31G (d) level.

**Table S2.** Energy analysis for the conformers of compound **1**.

| Conformation | E (Hartree) | E (kcal/mol) | Boltzmann Dist |
| --- | --- | --- | --- |
| **1a** | -892.7707239 | -560216.2183 | 24.13% |
| **1b** | -892.7693943 | -560215.384 | 5.9% |
| **1c** | -892.7681511 | -560214.6038 | 1.58% |
| **1d** | -892.7683197 | -560214.7096 | 1.59% |
| **1e** | -892.7714591 | -560216.6796 | 52.29% |
| **1f** | -892.769986 | -560215.3867 | 5.92% |
| **1g** | -892.769195 | -560215.2589 | 4.77% |
| **1h** | -892.7688252 | -560215.0268 | 3.23% |

**Table S3.** Cartesian coordinates for the low-energy optimized conformers of **1** at B3LYP/DGDZVP level of theory in CH_3_OH.

| Conformer **1e** | | Standard Orientation (Ångstroms) | | | |
| --- | --- | --- | --- | --- | --- |
| Center number | Atomic atom | Atomic type | X | Y | Z |
| 1 | 6 | 0 | -2.38468 | -2.94651 | 0.884525 |
| 2 | 6 | 0 | -2.56329 | -1.74331 | -0.01686 |
| 3 | 6 | 0 | -3.74842 | -0.81893 | 0.258655 |
| 4 | 6 | 0 | -3.38376 | 0.655258 | 0.011254 |
| 5 | 6 | 0 | -2.32928 | 1.204153 | 0.983911 |
| 6 | 6 | 0 | -2.01877 | 2.699114 | 0.794981 |
| 7 | 6 | 0 | -1.35371 | 3.084712 | -0.54018 |
| 8 | 6 | 0 | 0.035867 | 2.475296 | -0.80454 |
| 9 | 6 | 0 | 1.083618 | 2.82651 | 0.264656 |
| 10 | 6 | 0 | 2.542863 | 2.578822 | -0.15904 |
| 11 | 6 | 0 | 2.878729 | 1.154117 | -0.61945 |
| 12 | 6 | 0 | 2.794453 | 0.091804 | 0.464969 |
| 13 | 6 | 0 | 2.97356 | -1.39935 | 0.070557 |
| 14 | 6 | 0 | 1.556877 | -1.95401 | -0.21053 |
| 15 | 8 | 0 | 0.985238 | -1.29072 | -1.32332 |
| 16 | 8 | 0 | -1.8029 | -1.52021 | -0.94798 |
| 17 | 8 | 0 | 2.555061 | 0.383936 | 1.625786 |
| 18 | 6 | 0 | -4.92415 | -1.27288 | -0.63614 |
| 19 | 6 | 0 | 3.861945 | -1.59884 | -1.17137 |
| 20 | 6 | 0 | 3.56152 | -2.16679 | 1.268035 |
| 21 | 1 | 0 | -1.63876 | -3.62935 | 0.472288 |
| 22 | 1 | 0 | -3.33515 | -3.47253 | 1.032189 |
| 23 | 1 | 0 | -2.05437 | -2.60656 | 1.875157 |
| 24 | 1 | 0 | -4.04712 | -0.95128 | 1.308288 |
| 25 | 1 | 0 | -3.03317 | 0.750749 | -1.02216 |
| 26 | 1 | 0 | -4.30077 | 1.255222 | 0.094758 |
| 27 | 1 | 0 | -1.40256 | 0.623001 | 0.890178 |
| 28 | 1 | 0 | -2.68395 | 1.051305 | 2.013842 |
| 29 | 1 | 0 | -1.37874 | 3.028189 | 1.623415 |
| 30 | 1 | 0 | -2.95489 | 3.267818 | 0.894428 |
| 31 | 1 | 0 | -1.26303 | 4.180318 | -0.5645 |
| 32 | 1 | 0 | -2.02102 | 2.822778 | -1.37262 |
| 33 | 1 | 0 | -0.04048 | 1.385012 | -0.90858 |
| 34 | 1 | 0 | 0.380713 | 2.843953 | -1.78232 |
| 35 | 1 | 0 | 0.987997 | 3.892458 | 0.520541 |
| 36 | 1 | 0 | 0.884463 | 2.26905 | 1.1858 |
| 37 | 1 | 0 | 2.792224 | 3.261065 | -0.98397 |
| 38 | 1 | 0 | 3.199849 | 2.844976 | 0.67782 |
| 39 | 1 | 0 | 3.90055 | 1.130015 | -1.02631 |
| 40 | 1 | 0 | 2.233546 | 0.828835 | -1.44404 |
| 41 | 1 | 0 | 0.94437 | -1.81909 | 0.69467 |
| 42 | 1 | 0 | 1.645071 | -3.039 | -0.39695 |
| 43 | 1 | 0 | 0.033186 | -1.50104 | -1.34168 |
| 44 | 1 | 0 | -5.1957 | -2.32043 | -0.4608 |
| 45 | 1 | 0 | -5.80835 | -0.65955 | -0.43321 |
| 46 | 1 | 0 | -4.66322 | -1.16117 | -1.69392 |
| 47 | 1 | 0 | 4.865143 | -1.18447 | -1.01558 |
| 48 | 1 | 0 | 3.428068 | -1.1425 | -2.06374 |
| 49 | 1 | 0 | 3.977042 | -2.67111 | -1.37152 |
| 50 | 1 | 0 | 3.60078 | -3.2409 | 1.051795 |
| 51 | 1 | 0 | 2.966489 | -2.0049 | 2.169862 |
| 52 | 1 | 0 | 4.582333 | -1.82989 | 1.480397 |


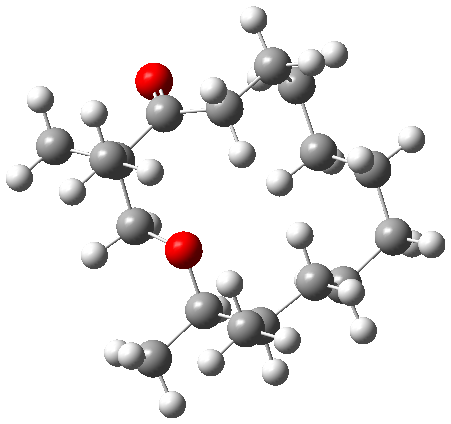

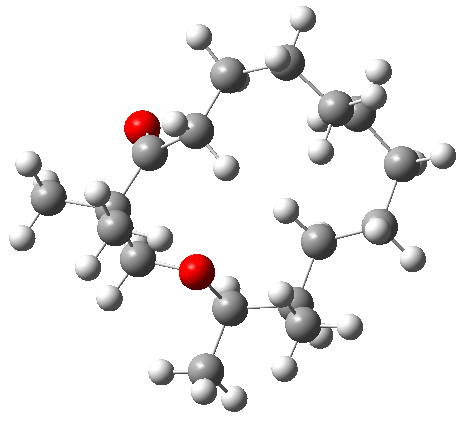


**2a 2b**

**Figure S34.** Optimized low-energy conformers of 12*R*, 13*S*-**2** at the B3LYP/6-31G (d) level.

**Table S4.** Energy analysis for the conformers of compound **2**.

| Conformation | E (Hartree) | E (kcal/mol) | Boltzmann Dist |
| --- | --- | --- | --- |
| **2a** | -817.5378603 | -513007.3782 | 1.67% |
| **2b** | -817.5417057 | -513009.7912 | 98.33% |

**Table S5.** Cartesian coordinates for the low-energy optimized conformers of **2** at PBEPBE/6-311+G level of theory in CH_3_OH.

| Conformer **2a** | | | Coordinates (Angstroms) | | |
| --- | --- | --- | --- | --- | --- |
| Center number | Atomic number | Atomic type | X | Y | Z |
| 1 | 6 | 0 | -2.13243 | 2.241114 | 1.004026 |
| 2 | 6 | 0 | -1.75985 | 2.925955 | -0.32755 |
| 3 | 6 | 0 | -0.32703 | 2.680402 | -0.82769 |
| 4 | 6 | 0 | -1.80454 | 0.743054 | 1.10541 |
| 5 | 6 | 0 | 0.781929 | 3.200899 | 0.097362 |
| 6 | 6 | 0 | 2.209185 | 2.898788 | -0.39637 |
| 7 | 6 | 0 | 2.548815 | 1.406501 | -0.58084 |
| 8 | 6 | 0 | 2.426144 | 0.579876 | 0.708481 |
| 9 | 6 | 0 | 2.720063 | -0.93226 | 0.5874 |
| 10 | 6 | 0 | 1.714102 | -1.68842 | -0.30949 |
| 11 | 8 | 0 | 0.39122 | -1.38388 | 0.169849 |
| 12 | 6 | 0 | -0.65689 | -1.67651 | -0.74179 |
| 13 | 6 | 0 | -2.51862 | -0.13951 | 0.091521 |
| 14 | 6 | 0 | -2.05795 | -1.61176 | -0.07315 |
| 15 | 6 | 0 | 1.955925 | -3.20341 | -0.29451 |
| 16 | 6 | 0 | 4.173071 | -1.18978 | 0.146934 |
| 17 | 6 | 0 | -2.02447 | -2.3302 | 1.292353 |
| 18 | 6 | 0 | -3.04345 | -2.32694 | -1.01572 |
| 19 | 8 | 0 | -3.44498 | 0.291648 | -0.57428 |
| 20 | 1 | 0 | -1.62638 | 2.745924 | 1.837204 |
| 21 | 1 | 0 | -3.20761 | 2.378622 | 1.167177 |
| 22 | 1 | 0 | -1.92321 | 4.00704 | -0.21171 |
| 23 | 1 | 0 | -2.46028 | 2.587658 | -1.09812 |
| 24 | 1 | 0 | -0.19134 | 1.604311 | -0.99605 |
| 25 | 1 | 0 | -0.21411 | 3.150195 | -1.81557 |
| 26 | 1 | 0 | -2.09335 | 0.378154 | 2.102893 |
| 27 | 1 | 0 | -0.72971 | 0.546084 | 1.038573 |
| 28 | 1 | 0 | 0.672449 | 4.289408 | 0.206867 |
| 29 | 1 | 0 | 0.649729 | 2.788391 | 1.105776 |
| 30 | 1 | 0 | 2.370232 | 3.412164 | -1.35523 |
| 31 | 1 | 0 | 2.926714 | 3.341411 | 0.309467 |
| 32 | 1 | 0 | 3.573423 | 1.33624 | -0.96937 |
| 33 | 1 | 0 | 1.9036 | 0.981838 | -1.36046 |
| 34 | 1 | 0 | 1.423006 | 0.695513 | 1.126565 |
| 35 | 1 | 0 | 3.117578 | 0.994278 | 1.457145 |
| 36 | 1 | 0 | 2.597706 | -1.35158 | 1.597471 |
| 37 | 1 | 0 | 1.803713 | -1.31951 | -1.34469 |
| 38 | 1 | 0 | -0.542 | -2.69414 | -1.13992 |
| 39 | 1 | 0 | -0.61724 | -0.98786 | -1.60295 |
| 40 | 1 | 0 | 1.942466 | -3.57852 | 0.735315 |
| 41 | 1 | 0 | 2.921489 | -3.45372 | -0.7435 |
| 42 | 1 | 0 | 1.191012 | -3.74273 | -0.86132 |
| 43 | 1 | 0 | 4.474603 | -2.23012 | 0.302026 |
| 44 | 1 | 0 | 4.325628 | -0.95284 | -0.91297 |
| 45 | 1 | 0 | 4.859673 | -0.56152 | 0.725851 |
| 46 | 1 | 0 | -3.01164 | -2.30218 | 1.768452 |
| 47 | 1 | 0 | -1.75175 | -3.38296 | 1.152168 |
| 48 | 1 | 0 | -1.29157 | -1.88776 | 1.969773 |
| 49 | 1 | 0 | -4.06604 | -2.2508 | -0.6365 |
| 50 | 1 | 0 | -3.03832 | -1.88204 | -2.01552 |
| 51 | 1 | 0 | -2.78241 | -3.38787 | -1.10166 |


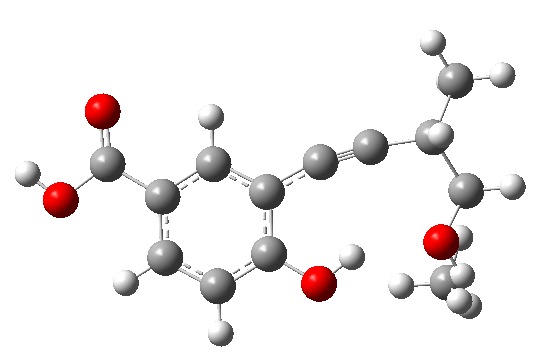

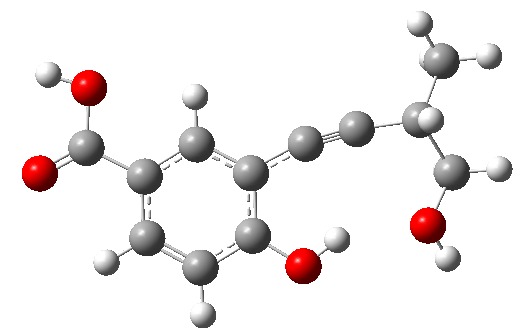

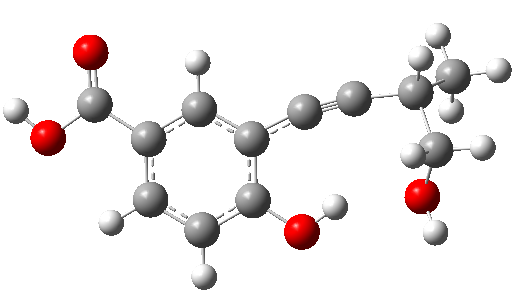


**4a 4b 4c**


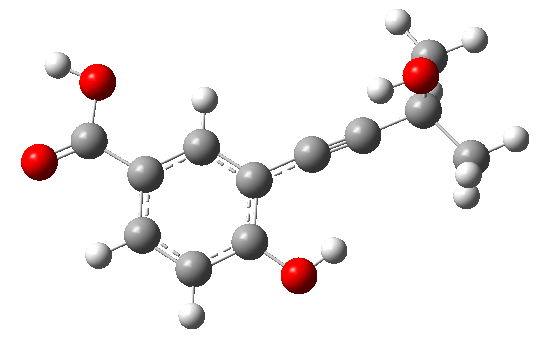

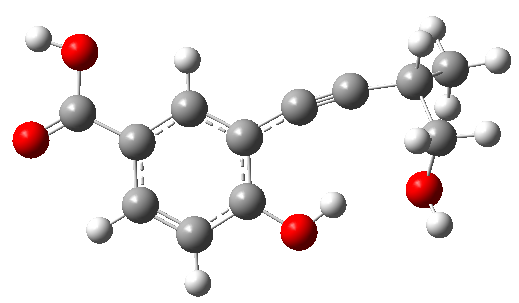

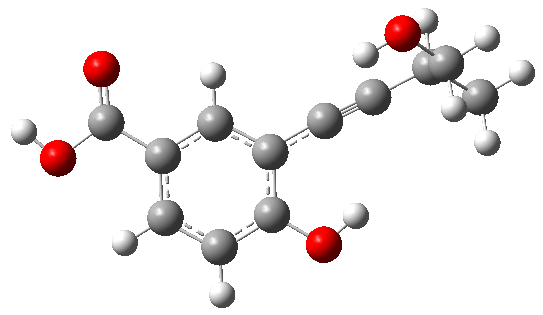


**4d 4e 4f**


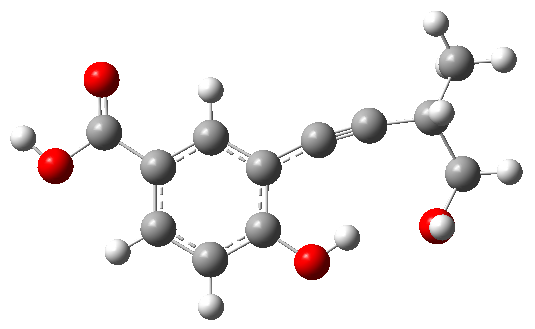

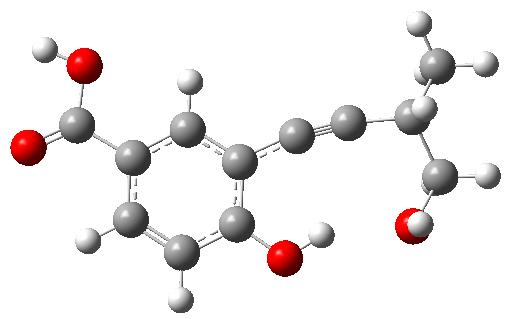


**4g 4h**

**Figure S35.** Optimized low-energy conformers of 12*S*-**4** at the B3LYP/6-31G (d) level.

**Table S6.** Energy analysis for the conformers of compound **4**.

| Conformation | E (Hartree) | E (kcal/mol) | Boltzmann Dist |
| --- | --- | --- | --- |
| **4a** | -765.3516783 | -480260.3977 | 13.63% |
| **4b** | -765.3517009 | -480260.4118 | 13.96% |
| **4c** | -765.3512314 | -480260.1172 | 8.49% |
| **4d** | -765.3500758 | -480259.3921 | 2.49% |
| **4e** | -765.3512621 | -480260.1365 | 8.77% |
| **4f** | -765.350082 | -480259.3960 | 2.51% |
| **4g** | -765.3522612 | -480260.7634 | 25.28% |
| **4h** | -765.3522457 | -480260.757 | 24.87% |

**Table S7.** Cartesian coordinates for the low-energy optimized conformers of **4** at B3LYP/6-31G level of theory in CH_3_OH.

| Conformer **4g** | | | Coordinates (Angstroms) | | |
| --- | --- | --- | --- | --- | --- |
| Center number | Atomic number | Atomic type | X | Y | Z |
| 1 | 6 | 0 | -0.58014 | 3.192558 | 0.704361 |
| 2 | 6 | 0 | -0.55732 | 2.925657 | 2.084819 |
| 3 | 6 | 0 | -0.2588 | 1.654731 | 2.554191 |
| 4 | 6 | 0 | 0.025286 | 0.617725 | 1.655345 |
| 5 | 6 | 0 | 0.003278 | 0.874148 | 0.255177 |
| 6 | 6 | 0 | -0.2997 | 2.161184 | -0.19715 |
| 7 | 6 | 0 | -0.89259 | 4.529749 | 0.156487 |
| 8 | 8 | 0 | -1.15043 | 5.455894 | 1.121221 |
| 9 | 8 | 0 | -0.92706 | 4.815963 | -1.02572 |
| 10 | 8 | 0 | 0.934219 | -3.19595 | 0.814761 |
| 11 | 6 | 0 | 0.401159 | -3.62894 | -0.43823 |
| 12 | 6 | 0 | 0.88238 | -2.6601 | -1.53491 |
| 13 | 6 | 0 | 0.567201 | -1.29602 | -1.10708 |
| 14 | 6 | 0 | 0.293191 | -0.22879 | -0.59899 |
| 15 | 6 | 0 | 0.271695 | -3.02738 | -2.89985 |
| 16 | 8 | 0 | 0.308739 | -0.59696 | 2.162204 |
| 17 | 1 | 0 | -0.77483 | 3.723516 | 2.786189 |
| 18 | 1 | 0 | -0.23617 | 1.436867 | 3.61705 |
| 19 | 1 | 0 | -0.32026 | 2.371329 | -1.26092 |
| 20 | 1 | 0 | -1.3385 | 6.282169 | 0.639612 |
| 21 | 1 | 0 | 0.617056 | -3.79735 | 1.505828 |
| 22 | 1 | 0 | 0.749599 | -4.64408 | -0.67821 |
| 23 | 1 | 0 | -0.69858 | -3.63446 | -0.41646 |
| 24 | 1 | 0 | 1.975609 | -2.76231 | -1.60005 |
| 25 | 1 | 0 | -0.82003 | -2.94457 | -2.87503 |
| 26 | 1 | 0 | 0.64348 | -2.35682 | -3.67968 |
| 27 | 1 | 0 | 0.537385 | -4.05417 | -3.17561 |
| 28 | 1 | 0 | 0.513113 | -1.24461 | 1.450639 |


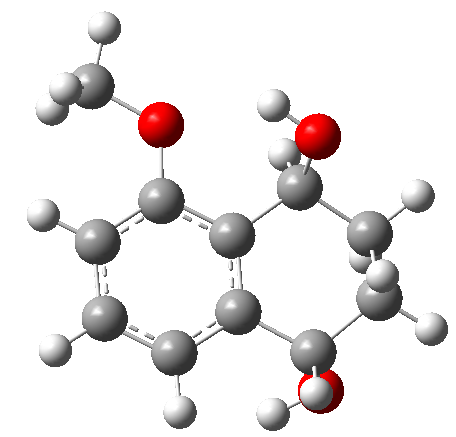

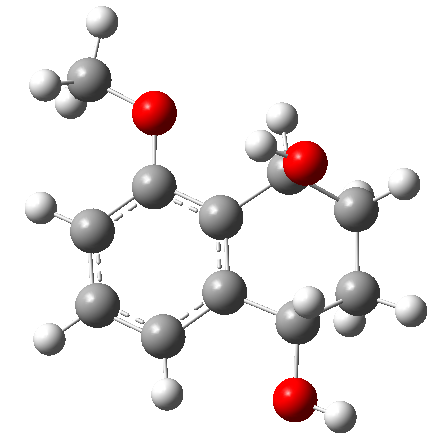

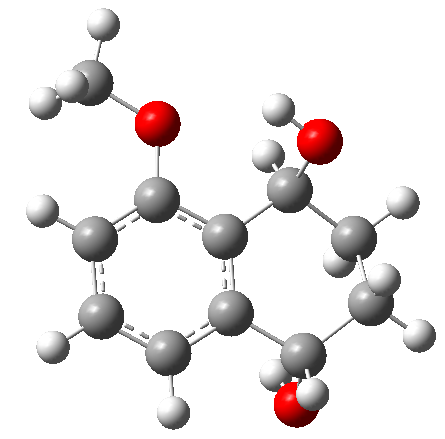


**5a 5b 5c**

**Figure S366.** Optimized low-energy conformers of 1*S*, 4*S***-5** at the B3LYP/6-31G (d) level.

**Table S8.** Energy analysis for the conformers of compound **5**.

| Conformation | E (Hartree) | E (kcal/mol) | Boltzmann Dist |
| --- | --- | --- | --- |
| **5a** | -653.2472244 | -409914.5277 | 57.0% |
| **5b** | -653.2416901 | -409911.0549 | 0.16% |
| **5c** | -653.2469549 | -409914.3586 | 42.84% |

**Table S9.** Cartesian coordinates for the low-energy optimized conformers of **5** at B3LYP/6-311G level of theory in CH_3_OH.

| Conformer **5a** | | | Coordinates (Angstroms) | | |
| --- | --- | --- | --- | --- | --- |
| Center number | Atomic number | Atomic type | X | Y | Z |
| 1 | 6 | 0 | -1.96293 | 1.511237 | -0.08477 |
| 2 | 6 | 0 | -1.03244 | 2.527885 | -0.31818 |
| 3 | 6 | 0 | 0.324188 | 2.234724 | -0.38547 |
| 4 | 6 | 0 | 0.779829 | 0.920048 | -0.19615 |
| 5 | 6 | 0 | -0.13401 | -0.10894 | 0.06175 |
| 6 | 6 | 0 | -1.5098 | 0.201963 | 0.084343 |
| 7 | 6 | 0 | 2.275531 | 0.645656 | -0.24623 |
| 8 | 6 | 0 | 2.579185 | -0.82093 | -0.5386 |
| 9 | 6 | 0 | 1.800922 | -1.71903 | 0.425606 |
| 10 | 6 | 0 | 0.290949 | -1.56163 | 0.254294 |
| 11 | 8 | 0 | -2.34387 | -0.87648 | 0.26701 |
| 12 | 8 | 0 | -0.09955 | -2.35168 | -0.87911 |
| 13 | 8 | 0 | 2.898072 | 0.956125 | 1.012936 |
| 14 | 6 | 0 | -3.7424 | -0.64668 | 0.362374 |
| 15 | 1 | 0 | -3.02063 | 1.746015 | -0.05491 |
| 16 | 1 | 0 | -1.38124 | 3.546627 | -0.46451 |
| 17 | 1 | 0 | 1.04124 | 3.023504 | -0.60462 |
| 18 | 1 | 0 | 2.716126 | 1.280831 | -1.03153 |
| 19 | 1 | 0 | 2.298682 | -1.04783 | -1.57394 |
| 20 | 1 | 0 | 3.658267 | -0.98194 | -0.43928 |
| 21 | 1 | 0 | 2.047227 | -2.77475 | 0.271829 |
| 22 | 1 | 0 | 2.075218 | -1.4558 | 1.45291 |
| 23 | 1 | 0 | -0.21266 | -1.95013 | 1.151497 |
| 24 | 1 | 0 | -1.06684 | -2.28022 | -0.93023 |
| 25 | 1 | 0 | 2.514047 | 1.788491 | 1.330087 |
| 26 | 1 | 0 | -4.14376 | -0.21499 | -0.56355 |
| 27 | 1 | 0 | -4.19565 | -1.62501 | 0.531769 |
| 28 | 1 | 0 | -3.98133 | 0.015533 | 1.203954 |


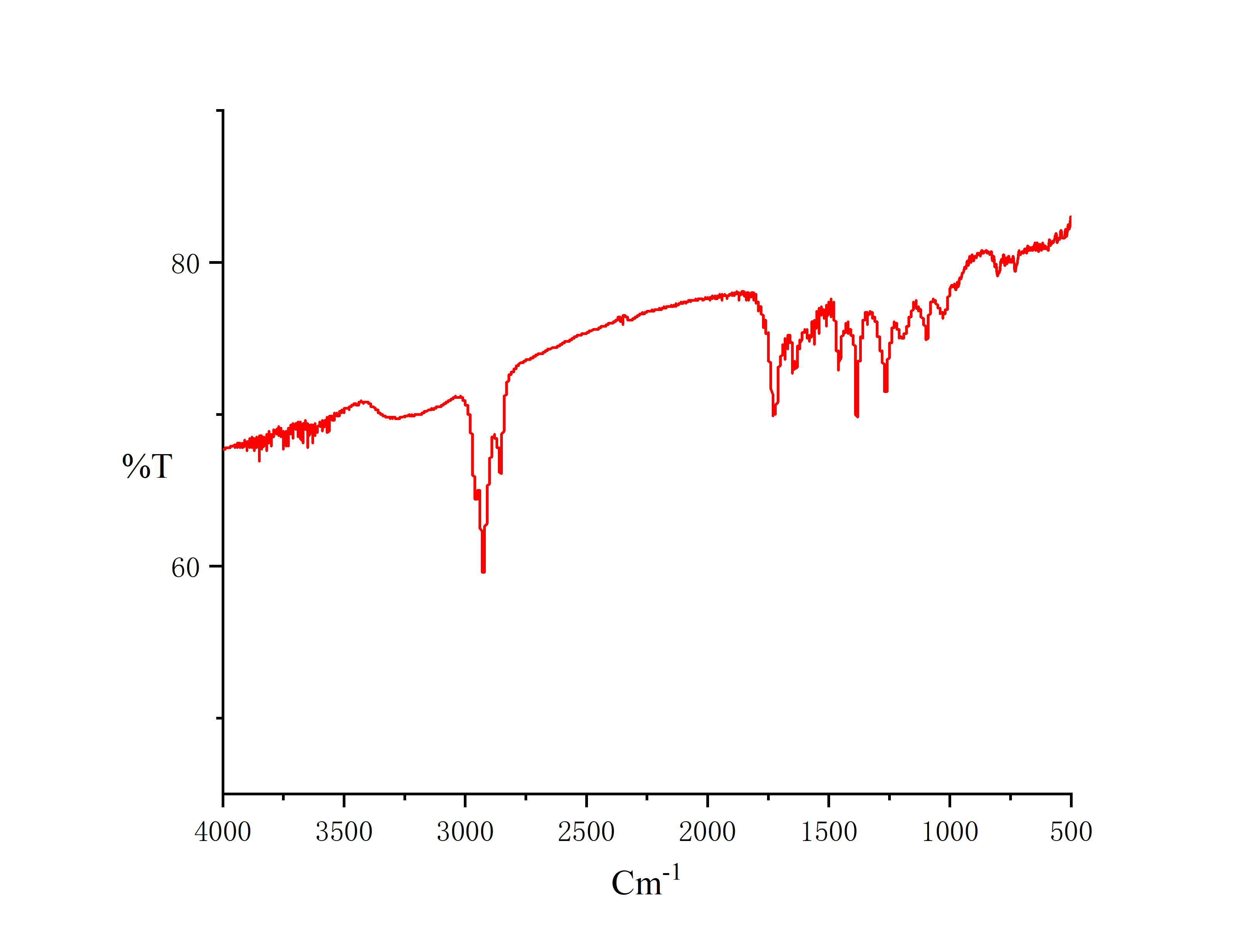


**Figure S37.** The IR spectrum of **2**.


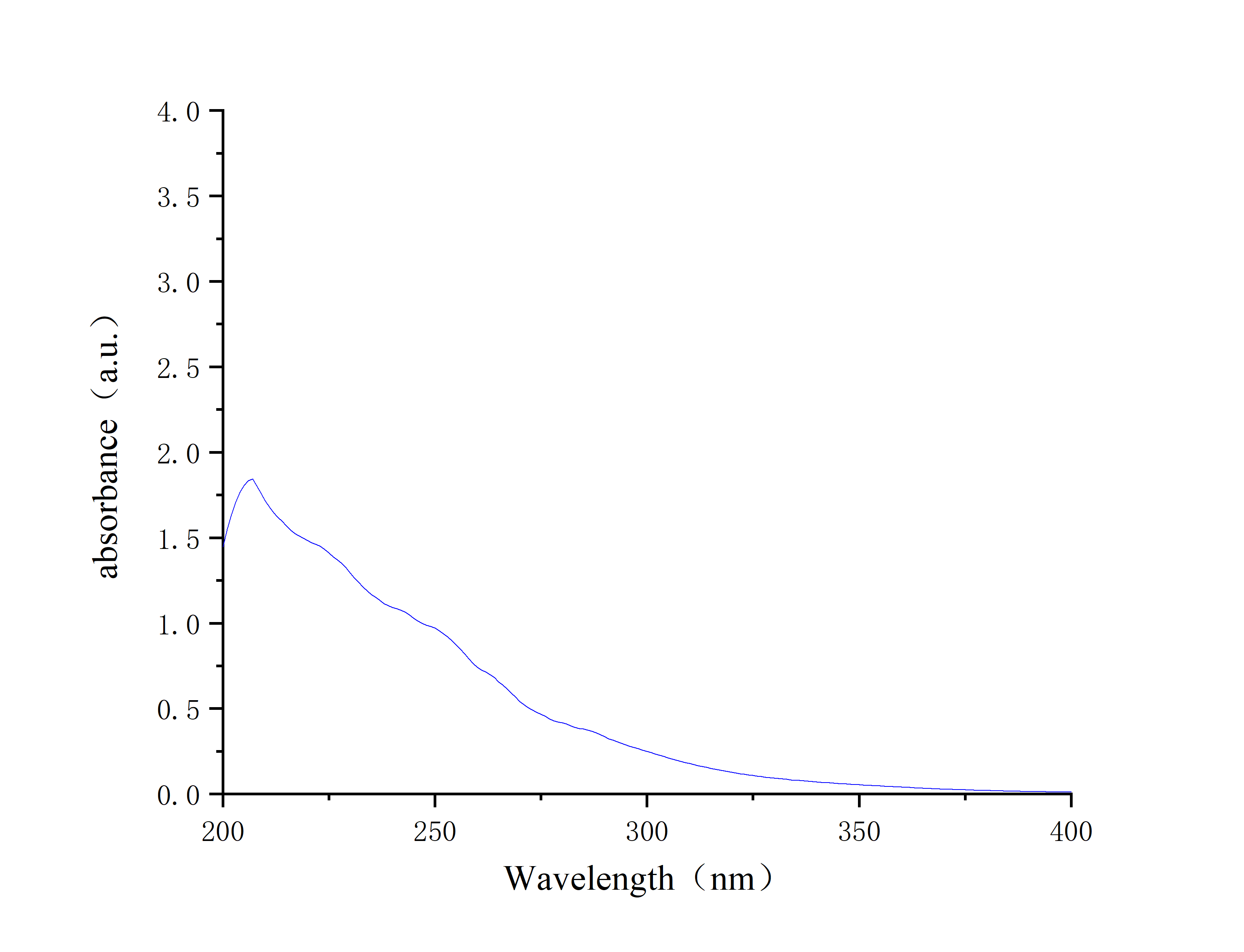


**Figure S38.** The UV spectrum of **2**.


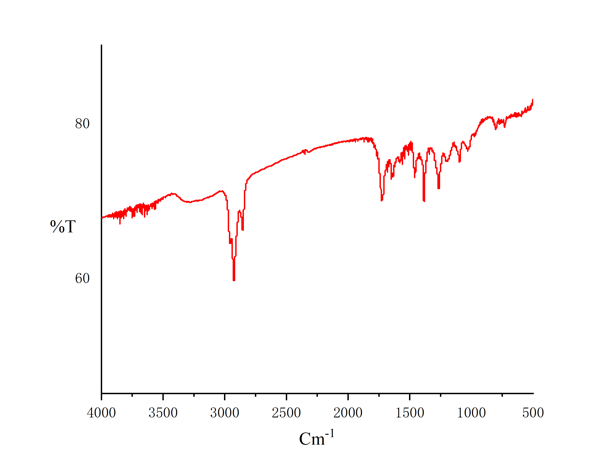


**Figure S39.** The IR spectrum of **6**.


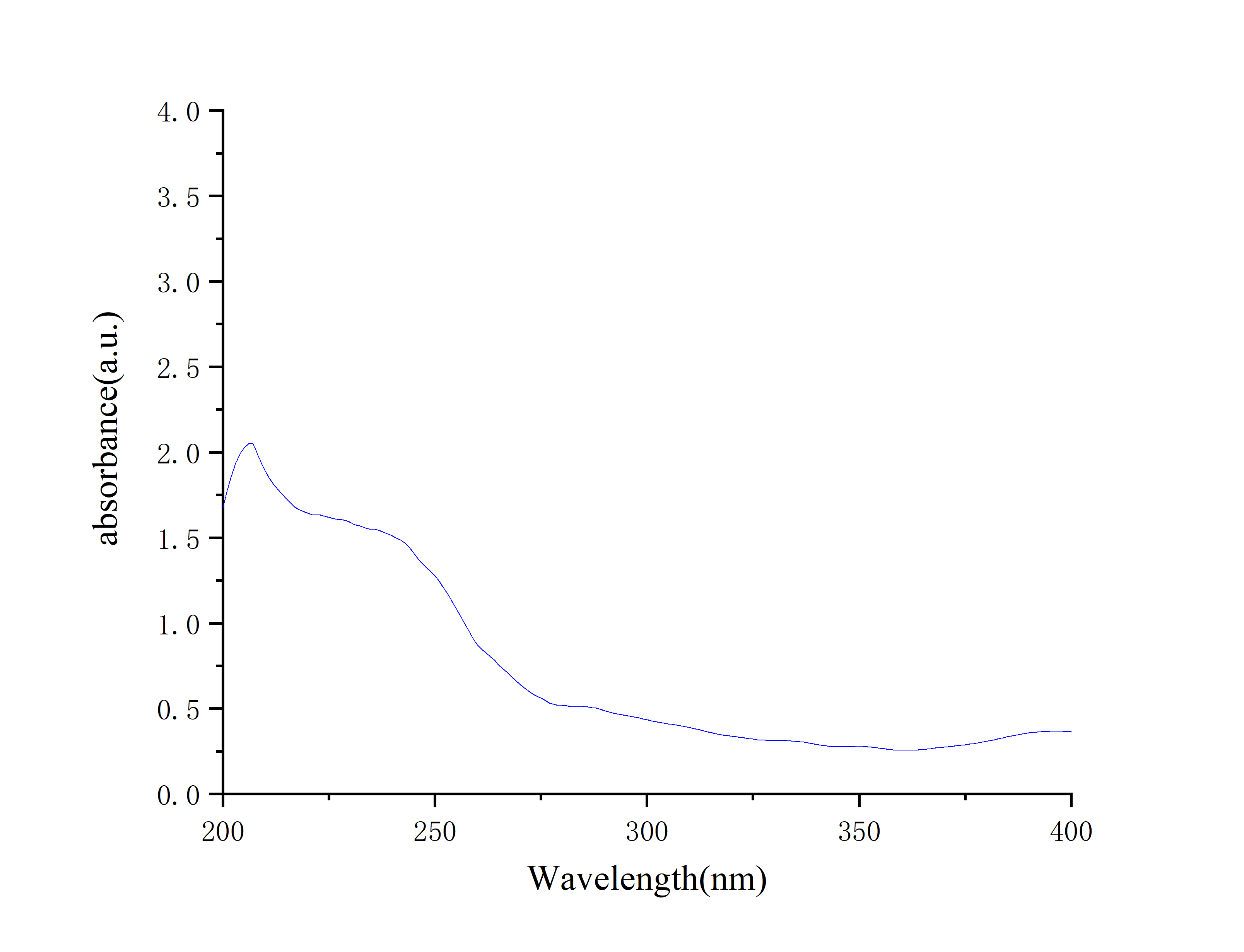


**Figure S40.** The UV spectrum of **6**.
